# PIN1 enhances SUMOylation of YAP1 and inhibits ferroptosis via autophagy-dependent degradation of ACSL4 in cervical cancer

**DOI:** 10.1038/s41420-026-03257-x

**Published:** 2026-07-22

**Authors:** Dan Liao, Lijuan Shi, Yumeng Cui, Cuifen Li, Yuhong Yao, Polin Feng, Huali Wang, Haohai Huang

**Affiliations:** 1 Department of Gynecology, The Affiliated Dongguan Songshan Lake Central Hospital, Guangdong Medical University, Dongguan, China; 2 Department of Gynecology, Dalian Women and Children’s Medical Center (Group), Dalian Medical University, Dalian, China; 3 Clinical Translational Medical Center, The Affiliated Dongguan Songshan Lake Central Hospital, Guangdong Medical University, Dongguan, China

**Keywords:** Cervical cancer, Tumour biomarkers, Cell death

## Abstract

Cervical cancer (CC) is a prevalent malignancy threatening women’s health. Due to its complex pathogenesis and tumor heterogeneity, it is urgent to thoroughly investigate its pathological mechanisms. Based on the TCGA database, the expression and survival analysis of PIN1 and YAP1 were assessed in CC. To verify their roles in regulating CC, stable knockdown and overexpression cell lines were constructed in CC cell lines. In vivo and in vitro experiments were conducted to verify that PIN1 regulates YAP1’s SUMOylation levels and induces ACSL4’s autophagic degradation. This research shows that PIN1 binds to YAP1, promoting its SUMOylation and causing autophagy-mediated degradation of ACSL4, enabling cervical cancer cells to resist ferroptosis. In vivo xenograft models confirm these findings. This study identifies PIN1 as a key redox-balance node and presents it as a potential therapeutic target for CC ferroptosis. Inhibiting PIN1 and YAP1 can inhibit tumor progression, offering a promising approach for treating aggressive CC.

## Introduction

Cervical cancer (CC) is one of the most common malignancies that poses a serious threat to women’s health. Due to its complex pathogenesis and significant tumor heterogeneity, in-depth exploration of its pathological mechanisms is particularly urgent [1]. Human papillomavirus (HPV) infection is the primary pathogenic cause of CC, with more than 90% of cases associated with high-risk HPV types [2, 3]. Although the advent of the HPV vaccine has significantly reduced the incidence of CC, and early diagnosis allows for effective management, advanced tumors often tend to metastasize and develop resistance to treatment, which frequently leads to poor patient prognosis [4, 5]. To address the challenges posed by advanced CC, ongoing research must be prioritized, focusing on the molecular mechanisms of tumor progression, metastasis, and drug resistance. The identification of new therapeutic targets and the screening of specific biomarkers will be crucial in overcoming this disease and improving patient outcomes.

Ferroptosis is a form of iron-dependent regulated cell death characterized by lipid peroxidation, and it has recently been recognized as a key regulatory factor in tumor progression and immune modulation [6–8]. Emerging evidence suggests that ferroptosis interacts with immune signaling pathways, leading to immunosuppression, antigen presentation defects, and remodeling of the tumor immune microenvironment in cervical cancer [9, 10]. This novel form of cell death, with its unique iron-dependent characteristics, exhibits significant regulatory effects across various tumor types. Ferroptosis is closely related not only to the survival ability of cancer cells but also to their invasion behavior and response to various therapeutic modalities [11–15]. Targeting ferroptosis-related molecules and pathways can selectively induce the death of cervical cancer cells, while alterations in the expression of ferroptosis-related genes provide promising biomarkers for prognostic evaluation. Numerous studies have shown that the regulatory network of ferroptosis in cervical cancer involves key molecules such as GPX4, SLC7A11, and ACSL4; imbalances in these pathways can lead to the accumulation of lipid peroxides, triggering cell death [16–19]. Recent research has found that activating ferroptosis through small-molecule compounds or genetic interventions can effectively inhibit tumor growth and enhance sensitivity to radiotherapy and chemotherapy. Additionally, there is a potential association between ferroptosis and the expression of the immune checkpoint PD-L1, suggesting a synergistic role in immunotherapy. A deeper understanding of the interaction mechanisms between ferroptosis and other modes of cell death will provide a theoretical basis for developing novel combination therapies.

PIN1 is a unique phosphorylation-dependent peptidyl-prolyl cis-trans isomerase that acts as a key regulatory factor in oncogenic signaling by catalyzing conformational changes in phosphorylated Ser/Thr-Pro motif proteins [20]. Its overexpression in various cancers drives tumorigenesis by simultaneously inactivating tumor suppressors and activating oncogenes, thereby promoting uncontrolled proliferation, evasion of cell death, genomic instability, epithelial-mesenchymal transition, metastasis, and treatment resistance. Clinically, PIN1 overexpression is associated with advanced disease stage, poor prognosis, and treatment failure, highlighting its value as a prognostic biomarker. PIN1 is overexpressed in more than 20 major human cancers, including triple-negative breast cancer, prostate cancer, hepatocellular carcinoma, and nasopharyngeal carcinoma [21–26]. This overexpression is caused by genomic amplification, oncogenic transcriptional activation, or post-translational stabilization that prevents its degradation. Additionally, a single amino acid substitution that restricts the regulatory motif of PIN1 interactions or its SUMOylation (SUMO-PIN1 auxiliary chromatin regulator) is sufficient to drive hyperaccumulation of RNF168, enhanced ubiquitin signaling, excessive recruitment of 53BP1, and radiosensitivity [27]. However, research on PIN1 in CC is still relatively limited.

Based on this background, we hypothesize that high expression of PIN1 promotes SUMO modification of YAP1, mediates the stability of YAP1, and thereby inhibits ferroptosis in cervical cancer by promoting autophagy-dependent degradation of ACSL4. In this study, we aim to clarify the effect of the PIN1/YAP1/ACSL4 pathway on ferroptosis in cervical cancer through in vivo and in vitro experiments.

## Results

### PIN1 significantly promotes the proliferation, migration, and invasion abilities of CC cells in vitro

**Fig. 1:**
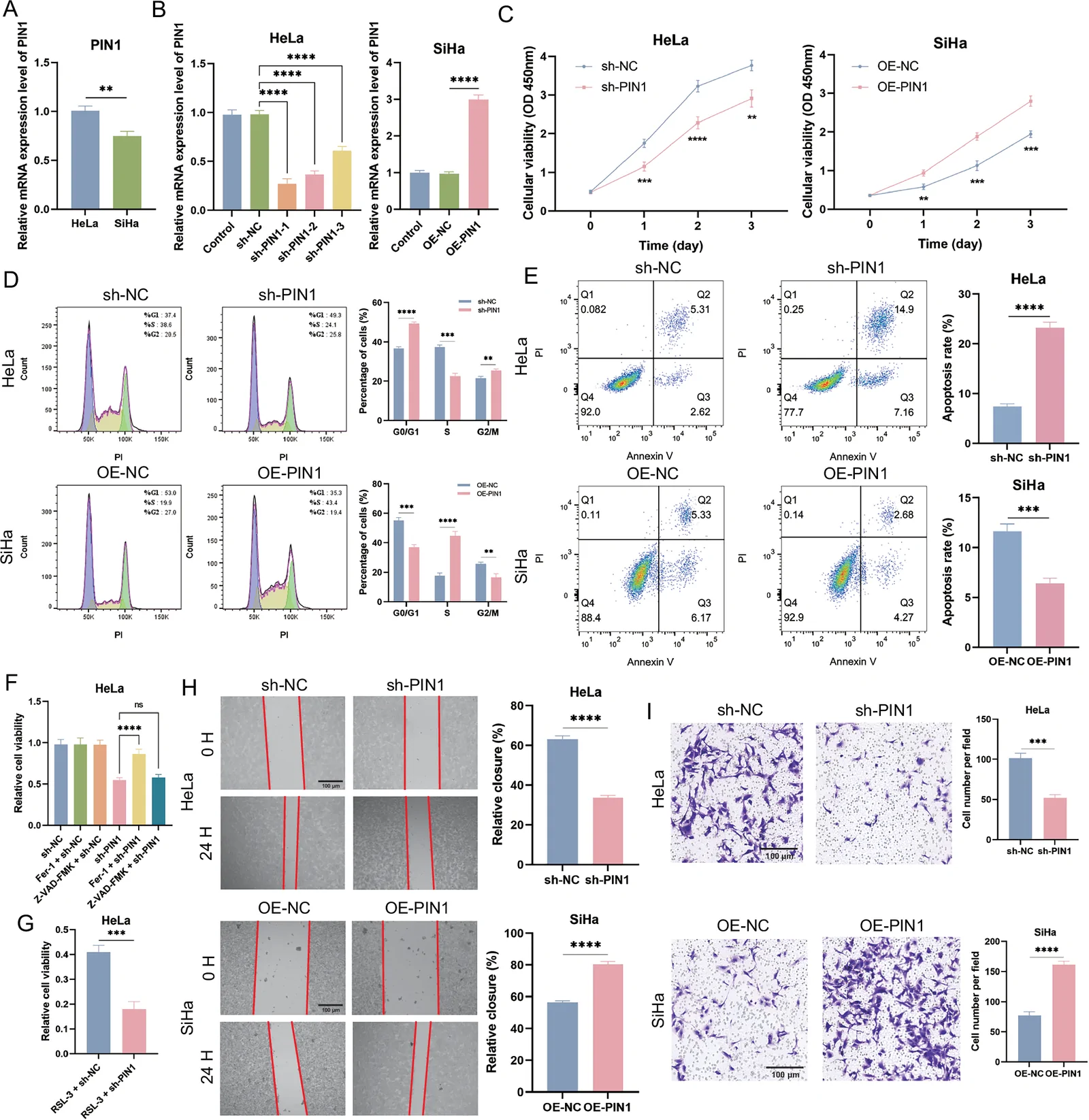
PIN1 significantly promotes the proliferation, migration, and invasion abilities of CC cells in vitro. **A** The results of qRT-PCR show the expression levels of PIN1 in cervical cancer cells. **B** qRT-PCR verifies the efficiency of constructing stable PIN1 knockdown and overexpression cell lines. **C** CCK8 assay results following PIN1 knockdown and overexpression. **D** Flow cytometry analysis of the cell cycle shows the changes in G0/G1, S, and G2/M phases after PIN1 knockdown and overexpression. **E** Death results after PIN1 knockdown and overexpression. **F** CCK-8 was used to detect the viability of HeLa cells treated with sh-NC, sh-PIN1, Fer-1 and Z-VAD-FMK, respectively. **G** CCK-8 was used to analyze the viability of PIN1-silenced HeLa cells treated with RSL3. **H** Cell scratch assay results (Bar = 100 μm). **I** Transwell assay results (Bar = 100 μm). **P < 0.01, ***P < 0.001, ****P < 0.0001, ns not significant.

To investigate the expression of PIN1 in CC, we first utilized bioinformatics analysis, which revealed that PIN1 is significantly overexpressed in CC (Fig. S1A). Kaplan-Meier survival curve analysis suggested a close correlation between PIN1 expression and poor prognosis (Fig. S1B). The ROC curve with AUC indicated PIN1 may serve as a potential prognosis biomarker for CC (Fig. S1C). qRT-PCR experiments revealed that the expression level of PIN1 is higher in the HeLa cell line compared to the SiHa cell line (Fig. 1A). This observation provided a crucial foundation for our subsequent experimental designs. Building on this initial finding, we successfully established a stable PIN1 knockdown cell line within the HeLa cells, effectively reducing the expression of PIN1. Conversely, we also developed a PIN1 overexpression cell line in the SiHa cells, thereby enhancing the levels of PIN1 in these cells (Fig. 1B). These engineered cell lines were then utilized for a series of phenotypic experiments to elucidate the functional roles of PIN1 in CC cells. The results obtained from the CCK-8 assays and cell cycle analysis demonstrated that the knockout of PIN1 led to a significant reduction in the proliferative ability of the CC cells. This indicates that PIN1 plays a critical role in sustaining the growth and proliferation of these cancer cells. On the other hand, the overexpression of PIN1 in the SiHa cells significantly promoted their proliferative capacity, further corroborating the pro-proliferative role of PIN1 in CC (Fig. 1C, D). Additionally, flow cytometry experiments revealed that the downregulation of PIN1 expression significantly enhanced the death of the CC cells. This suggests that PIN1 may inhibit death, thereby allowing cancer cells to evade programmed cell death. In contrast, the overexpression of PIN1 notably inhibited the death process, reinforcing the idea that PIN1 supports the survival of cancer cells (Fig. 1E). To investigate whether the cell death induced by PIN1 knockdown in HeLa cells is apoptosis or ferroptosis, we treated HeLa cells with the apoptosis inhibitor Z-VAD-FMK (MCE, HY-16658B) and the ferroptosis inhibitor Fer-1 (MCE, HY-100579). Firstly, PIN1 knockdown promoted cell death, and this effect was inhibited by Fer-1, while the apoptosis inhibitor Z-VAD-FMK failed to reverse the cell death induced by PIN1 inhibition (Fig. 1F). To determine whether PIN1 could mediate ferroptosis in CC, we silenced PIN1 and synchronously treated the cells with the ferroptosis inducer, RSL3. The results showed that the abrogation of PIN1 enhanced RSL3-induced ferroptosis (Fig. 1G). These results indicated that cell death mediated by knockdown of PIN1 was mainly through ferroptosis. Moreover, wound healing assays and Transwell assays provided insights into the effects of PIN1 on the migratory and invasive abilities of cervical cancer cells. The results indicated that the knockout of PIN1 markedly weakened these abilities, implying that PIN1 is essential for the mobility and invasiveness of CC cells. Conversely, the overexpression of PIN1 exhibited the opposite effect, significantly enhancing the migratory and invasive capabilities of the cells (Fig. 1H, I). Collectively, these comprehensive experimental results suggest that PIN1 functions as an oncogene in the context of CC. The data imply that PIN1 promotes cancer cell proliferation, survival, migration, and invasion, contributing to disease malignancy. Consequently, PIN1 may be a valuable prognostic biomarker for cervical cancer, helping predict outcomes and develop targeted therapies.

### PIN1 inhibits ferroptosis and promotes the progression of cervical cancer

**Fig. 2:**
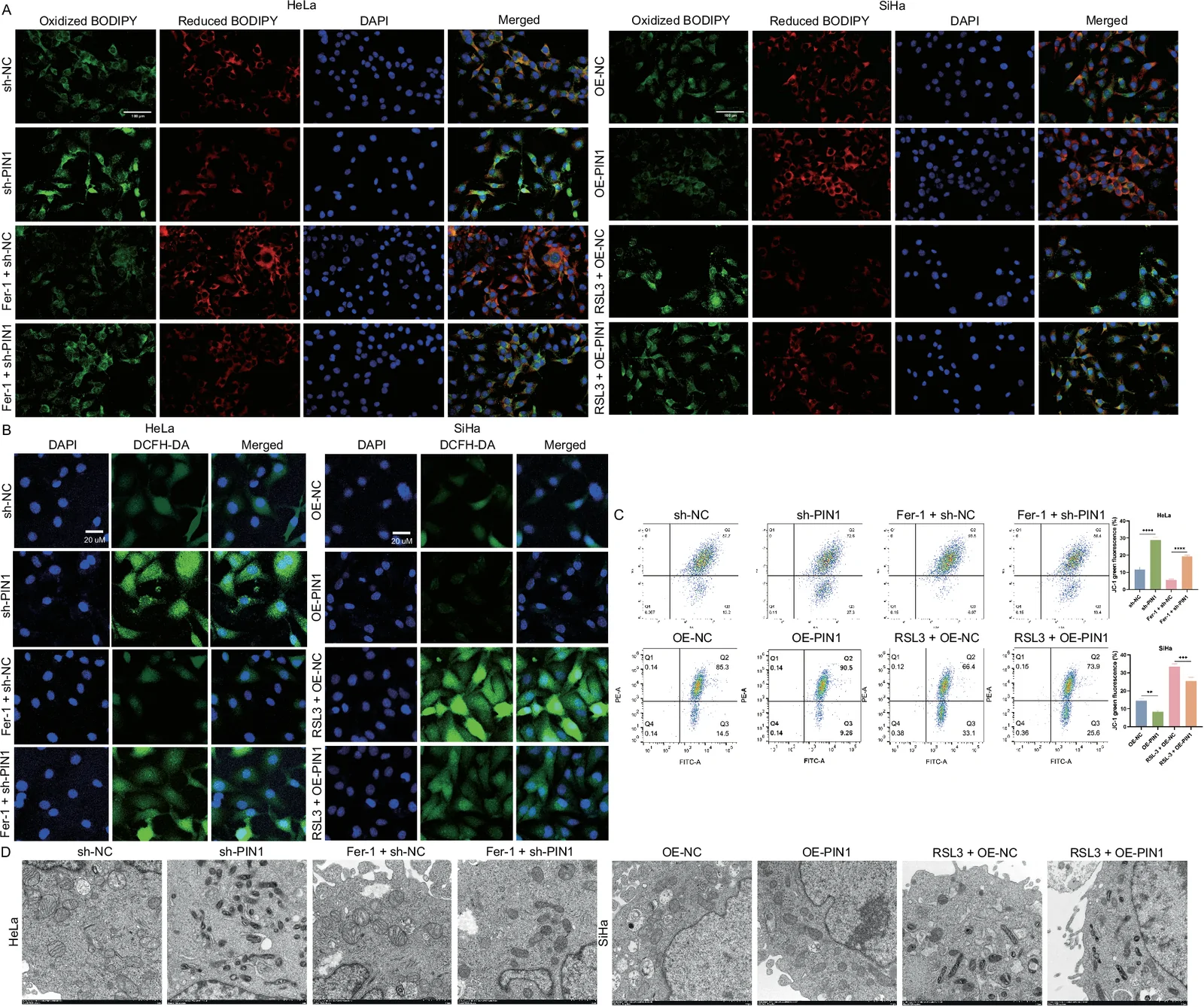
PIN1 inhibits ferroptosis and promotes the progression of cervical cancer. **A** C11-BODIPY 581/591 probe-stained images after PIN1 knockdown and overexpression, as well as after the addition of Fer-1 in the knockdown group and RSL3 in the overexpression group (Bar = 100 μm). **B** knockdown and overexpression, as well as after the addition of Fer-1 in the knockdown group and RSL3 in the overexpression group (Bar = 20 μm). **C** JC-1 flow analysis results after PIN1 knockdown and overexpression, as well as after the addition of Fer-1 in the knockdown group and RSL3 in the overexpression group. **D** Electron microscopy observations of mitochondrial structural integrity after PIN1 knockdown and overexpression, as well as after the addition of Fer-1 in the knockdown group and RSL3 in the overexpression group (Bar = 1 μm). **P < 0.01, ***P < 0.001, ****P < 0.0001.

To investigate the relationship between PIN1 and ferroptosis in CC, we introduced the ferroptosis inhibitor Fer-1 into PIN1 knockdown stable HeLa cell lines and the ferroptosis agonist RSL3 into PIN1 overexpression stable SiHa cell lines. We first performed lipid ROS detection via C11-BODIPY, and the results showed increased green fluorescence in HeLa cells after the knockdown of PIN1, indicating greater lipid ROS production in these cells. Treatment with Fer-1 reduced green fluorescence, effectively reversing the effects of PIN1 silencing. In contrast, overexpression of PIN1 resulted in decreased green fluorescence, while treatment with RSL3 caused increased green fluorescence (Fig. 2A). Staining images obtained using the DCFH-DA probe showed that the knockout of PIN1 significantly increased ROS production. Treatment with Fer-1 reduced ROS levels, effectively reversing the effects of PIN1 silencing. Conversely, overexpression of PIN1 markedly reduced ROS generation, while treatment with RSL3 significantly diminished the inhibitory effect of PIN1 on ROS (Fig. 2B). Subsequent JC-1 flow cytometry results indicated that, following PIN1 gene knockout, the percentage of green fluorescence significantly increased, whereas overexpression of PIN1 was associated with an increase in red fluorescence. After adding Fer-1 and RSL3 to the knockout and overexpression groups, respectively, these results were reversed, indicating that PIN1 expression has a significant impact on mitochondrial membrane potential (Fig. 2C). Mitochondrial structures were also examined via electron microscopy, revealing that silencing PIN1 led to significant mitochondrial shrinkage and loss of cristae, indicative of unhealthy mitochondria. This phenomenon could be significantly reversed with Fer-1 treatment. In contrast, overexpression of PIN1 resulted in clearer mitochondrial structures, while treatment with RSL3 caused mitochondrial shrinkage (Fig. 2D). These findings demonstrate that PIN1 is crucial for maintaining mitochondrial structural integrity and stabilizing membrane potential, and that PIN1 can inhibit the ferroptosis process, thereby promoting the occurrence and progression of CC.

### PIN1 regulates SUMOylation to promote the expression of YAP1, thereby facilitating the autophagy-dependent degradation of ACSL4

**Fig. 3:**
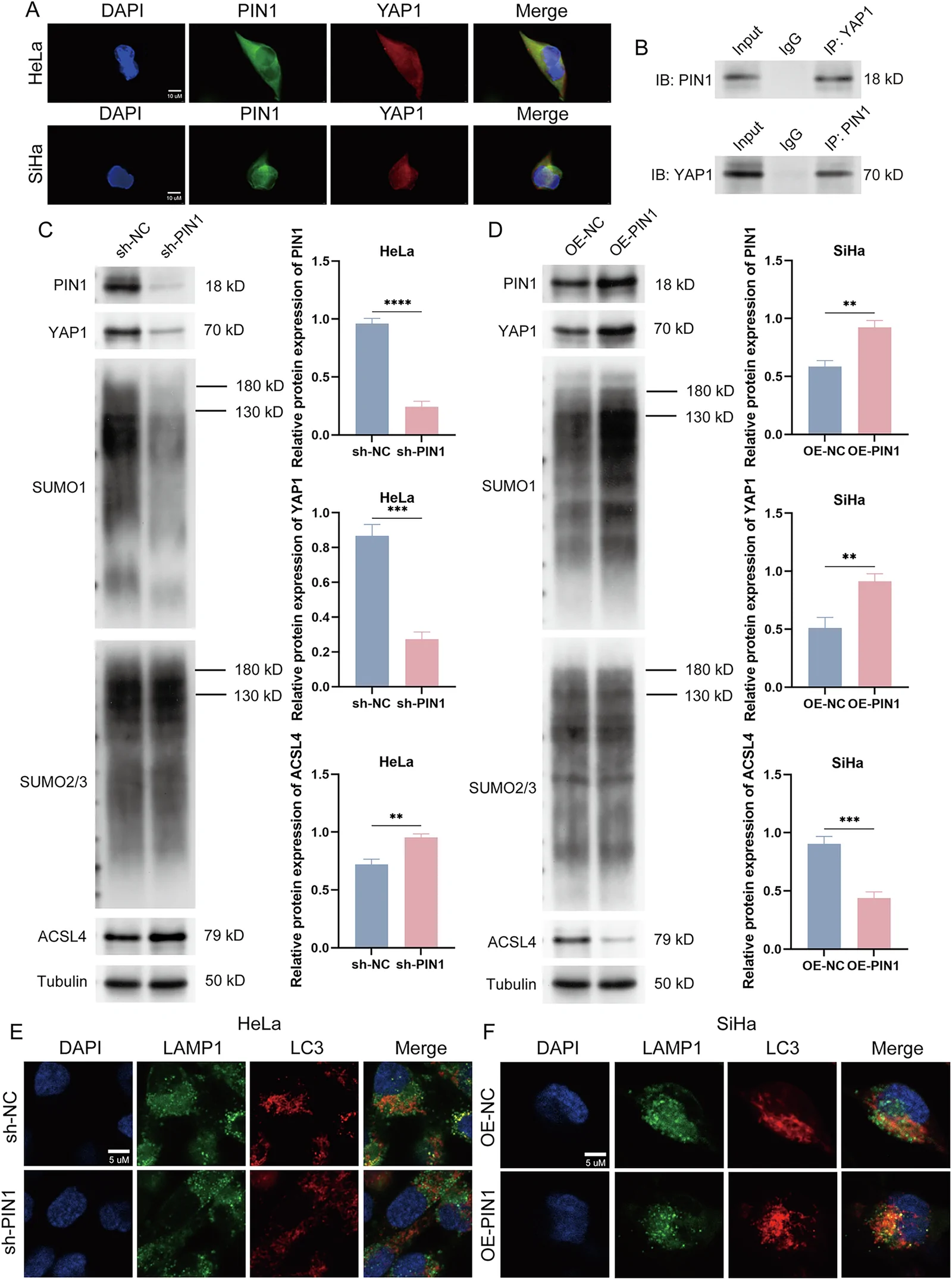
PIN1 regulates SUMOylation to promote the expression of YAP1, thereby facilitating the autophagy-dependent degradation of ACSL4. **A** Fluorescence co-localization result of PIN1 and YAP1 (Bar = 10 μm). **B** Co-IP experiments validate the ability of PIN1 to interact with YAP1. **C** Western blot results showing the effects of PIN1 knockdown on YAP1 expression, as well as on YAP1 SUMOylation and ACSL4 expression. **D** Western blot results showing the effects of PIN1 overexpression on YAP1 expression, as well as on YAP1 SUMOylation and ACSL4 expression. **E** Immunofluorescence experiments validate the effects of PIN1 knockdown on the expression of autophagy and lysosomal markers LAMP1 and LC3 (Bar = 5 μm). **F** Immunofluorescence experiments validate the effects of PIN1 overexpression on the expression of autophagy and lysosomal markers LAMP1 and LC3 (Bar = 5 μm). **P < 0.01, ***P < 0.001, ****P < 0.0001.

**Fig. 4:**
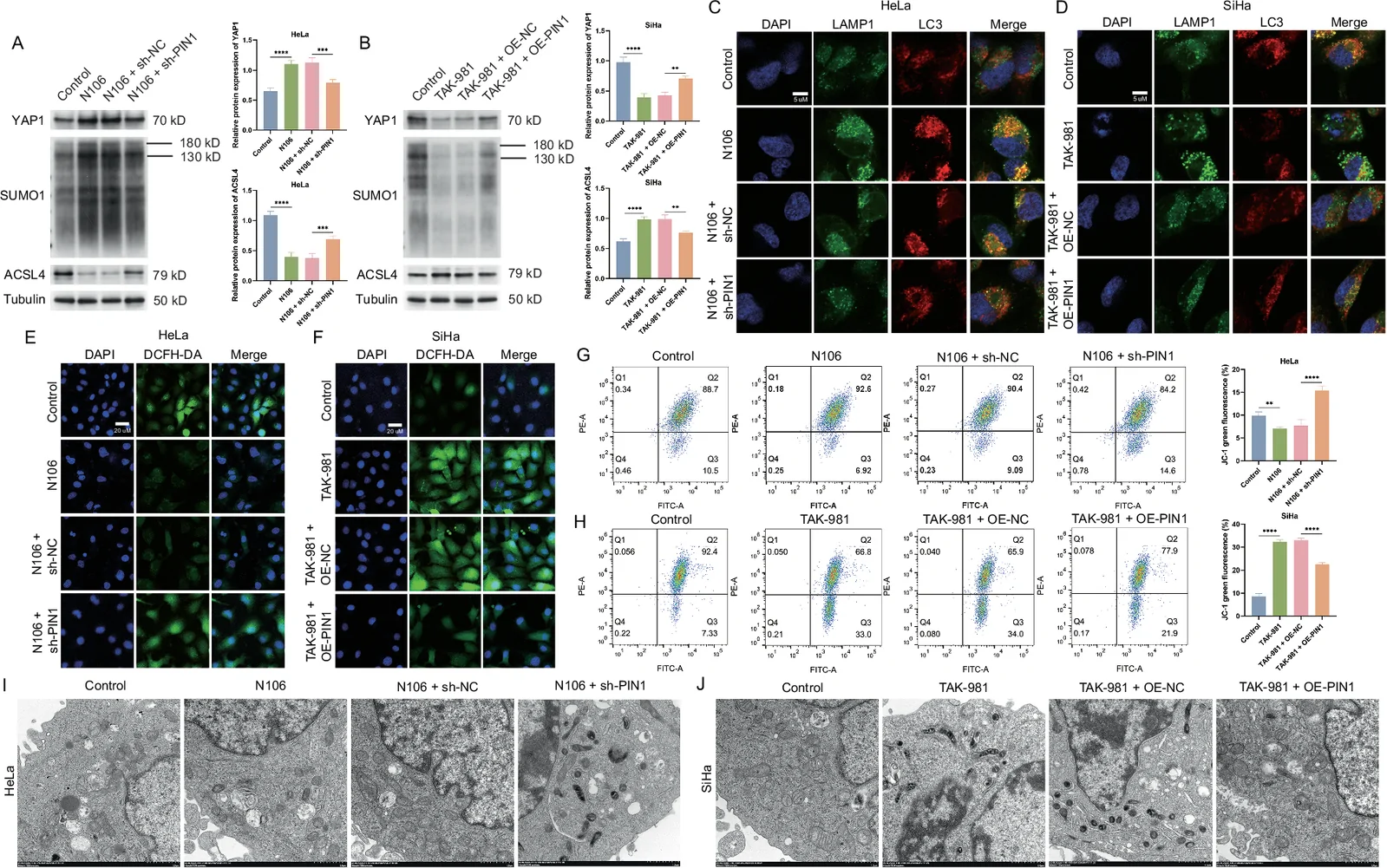
PIN1 promotes the malignant phenotype of CC cells by regulating the SUMOylation of YAP1. **A** Western blot experiments validating the effects of PIN1 knockdown and the addition of the SUMOylation activator N106 on the SUMOylation of YAP1. **B** Western blot experiments validating the effects of PIN1 overexpression and the addition of the SUMOylation inhibitor TAK-981 on the SUMOylation of YAP1. **C** The effects of PIN1 knockdown and the addition of the SUMOylation activator N106 on the fluorescence co-localization of LAMP1 and LC3 (Bar = 5 μm). **D** The effects of PIN1 overexpression and the addition of the SUMOylation inhibitor TAK-981 on the fluorescence co-localization of LAMP1 and LC3 (Bar = 5 μm). **E** The effects of PIN1 knockdown and the addition of the SUMOylation activator N106 on ROS accumulation (Bar = 20 μm). **F** The effects of PIN1 overexpression and the addition of the SUMOylation inhibitor TAK-981 on ROS accumulation (Bar = 20 μm). **G** JC-1 flow analysis results after PIN1 knockdown and the addition of the SUMOylation activator N106. **H** JC-1 flow analysis results after PIN1 overexpression and the addition of the SUMOylation inhibitor TAK-981. **I** Electron microscopy observations of mitochondrial structure after PIN1 knockdown and the addition of the SUMOylation activator N106 (Bar = 1 μm). **J** Electron microscopy observations of mitochondrial structure after PIN1 overexpression and the addition of the SUMOylation inhibitor TAK-981 (Bar = 1 μm). **P < 0.01, ***P < 0.001, ****P < 0.0001.

To delve deeper into the intricate mechanism by which PIN1 facilitates the progression of CC, we meticulously investigated the interplay between PIN1, YAP1, and various autophagy-related markers. Initially, we found that PIN1 and YAP1 demonstrated a strong correlation based on correlation analysis (Fig. S1D). And the molecular docking analysis further indicated the correlation (Fig. S1E). Several studies have indicated that YAP1 could serve as a biomarker for CC. We used some bioinformatics analysis to evaluate the function of YAP1, which indicated that YAP1 was significantly overexpressed in CC (Fig. S1F). KM and ROC curves demonstrated that YAP1 is extremely associated with a worse situation of CC (Fig. S1G, H). Our immunofluorescence experiments revealed a striking co-localization of PIN1 and YAP1 within CC cell lines (Fig. 3A), and subsequent co-immunoprecipitation assays definitively confirmed that PIN1 engages in direct interaction with YAP1 (Fig. 3B). The knockdown of PIN1 resulted in a significant inhibition of YAP1 expression, a notable reduction in its SUMOylation capacity (SUMO1), and a pronounced promotion of ACSL4 expression. Conversely, the overexpression of PIN1 led to a substantial increase in both YAP1 expression and its SUMOylation capability (SUMO1), while concurrently inhibiting ACSL4 expression (Fig. 3C, D). These findings collectively indicate that PIN1 plays a pivotal role in enhancing the SUMOylation ability and expression of YAP1. This post-translational modification serves to stabilize YAP1 and amplify its transcriptional activity, thereby leading to the upregulation of critical autophagy-related genes. In addition, to further explore the relationship between the PIN1-YAP1 axis and ferroptosis, we found that YAP1 was significantly associated with the expression of ACSL4 rather than GPX4 and SLC7A11 (Fig. S1I–K). Subsequently, we probed the interaction of PIN1 with autophagosomes and lysosomes using immunofluorescence techniques. Our results demonstrated that the knockdown of PIN1 markedly diminished the co-localization of PIN1 with LAMP1 and LC3, whereas the overexpression of PIN1 significantly augmented their co-localization (Fig. 3E). To further corroborate the regulation of YAP1 SUMOylation by PIN1, we introduced a SUMOylation activator, N106, and a suppressor, TAK-981, into the PIN1 knockdown and overexpression experimental groups. Western blot assays revealed that N106 significantly bolstered the expression levels of YAP1 and SUMO1 and decreased ACSL4 expression, whereas the knockdown of PIN1 substantially weakened the efficacy of N106. On the other hand, TAK-981 markedly inhibited the expression levels of YAP1 and SUMO1, and increased ACSL4 expression, although the overexpression of PIN1 partially reversed the inhibitory effect of TAK-981 (Fig. 4A, B). We also evaluated the co-localization abilities of autophagy and lysosome-related markers in the presence of SUMOylation inhibitors and activators. Immunofluorescence results indicated that N106 significantly enhanced the co-localization with LAMP1 and LC3, whereas TAK-981 exhibited an opposing effect (Fig. 4C, D). Additionally, ROS immunofluorescence assays showed that N106 significantly reduced ROS levels, whereas TAK-981 substantially increased ROS levels (Fig. 4E, F). Subsequent JC-1 flow cytometry analyses revealed that PIN1 gene knockout led to a significant increase in the percentage of green fluorescence, indicative of mitochondrial membrane potential disruption, while overexpression of PIN1 was associated with an increase in red fluorescence, suggesting mitochondrial membrane potential stabilization (Fig. 4G, H). Electron microscopy analysis of mitochondrial structures further illuminated that treatment with N106 resulted in more intact and well-organized mitochondria and cristae, whereas TAK-981 treatment caused noticeable mitochondrial shrinkage and cristae loss, indicative of unhealthy mitochondrial morphology (Fig. 4I, J). This regulatory axis underscores the mechanistic relationship between PIN1-mediated signaling and the cellular antioxidant stress capabilities in cervical cancer cells. Inhibiting ferroptosis through this pathway aids tumor survival under metabolic and oxidative stress conditions, thereby highlighting the potential of PIN1 as a strategic therapeutic target. Disrupting the PIN1-YAP1 interaction can effectively restore ACSL4 levels and render cervical cancer cells more sensitive to ferritin deposition inducers, offering a novel strategy to overcome treatment resistance. By inhibiting PIN1, YAP1 fails to stabilize ACSL4, leading to a diminished ACSL4-mediated lipid repair function, increased accumulation of lipid peroxides, and ultimately triggering ferroptotic cell death. This comprehensive understanding of the PIN1-YAP1 axis provides critical insights into the development of targeted therapies for cervical cancer, potentially paving the way for more effective treatment strategies.

### Effect of YAP1 on cervical cancer cell activity and its regulation of ACSL4

**Fig. 5:**
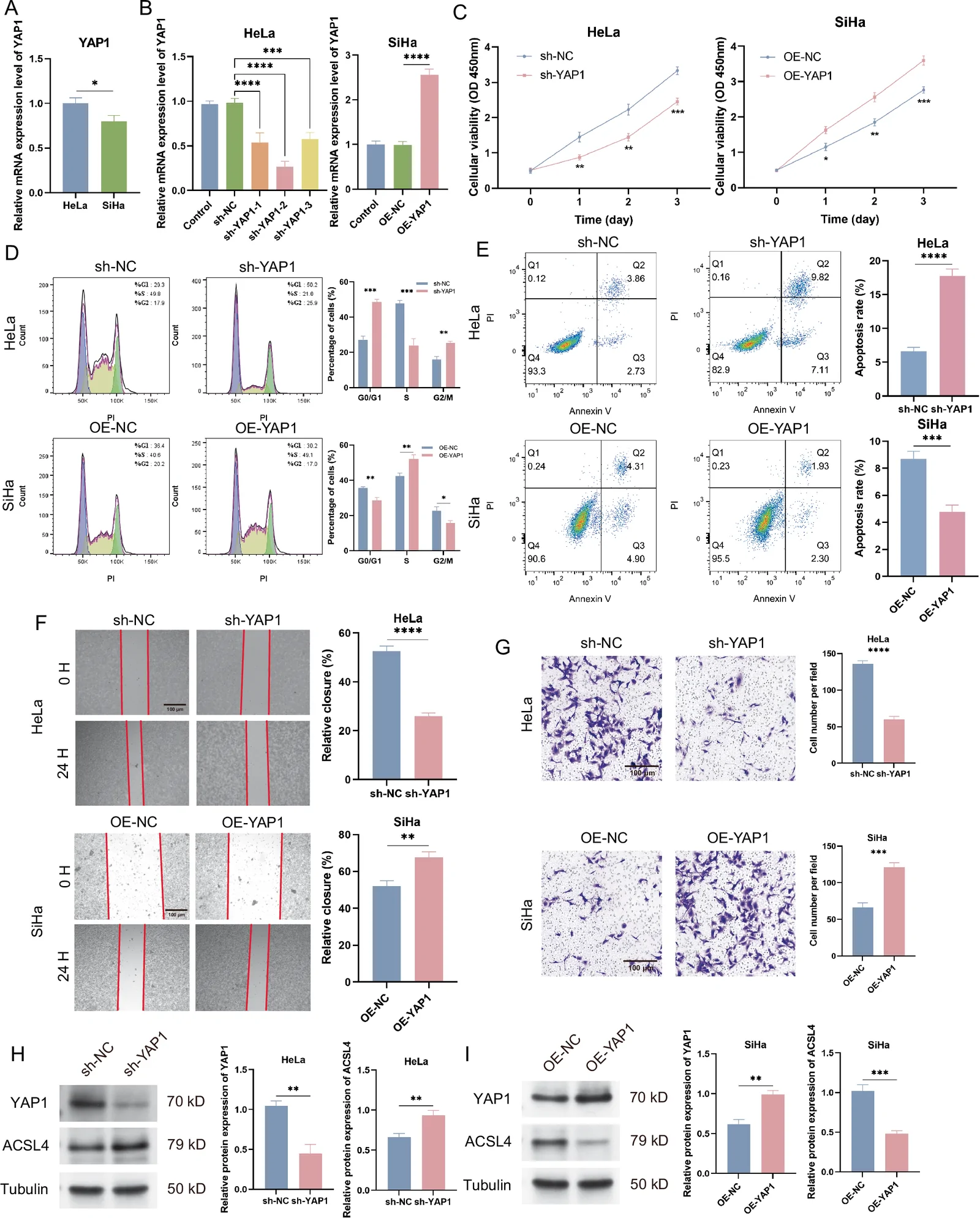
Effect of YAP1 on cervical cancer cell activity and its regulation of ACSL4. **A** The results of qRT-PCR show the expression levels of YAP1 in cervical cancer cells. **B** qRT-PCR verifies the efficiency of constructing stable YAP1 knockdown and overexpression cell lines. **C** CCK8 assay results following YAP1 knockdown and overexpression. **D** Flow cytometry analysis of the cell cycle shows changes in G0/G1, S, and G2/M phases after YAP1 knockdown and overexpression. **E** Death results after YAP1 knockdown and overexpression. **F** Cell scratch assay results (Bar = 100 μm). **G** Transwell assay results (Bar = 100 μm). **H** Western blot results showing the promotion of ACSL4 expression after YAP1 knockdown. **I** Western blot results showing the inhibition of ACSL4 expression after YAP1 overexpression. *P < 0.05, **P < 0.01, ***P < 0.001, ****P < 0.0001.

To further investigate the role of YAP1 in the progression of CC, we first conducted qRT-PCR experiments, which revealed that the expression level of YAP1 is higher in the HeLa cell line compared to the SiHa cell line (Fig. 5A). Consequently, we constructed stable YAP1 knockdown cell lines using a shRNA interference strategy in HeLa cells, while also developing stable YAP1 overexpression lines in SiHa cells. We found that sh-YAP1-2 exhibited the highest knockdown efficiency and thus used it for subsequent phenotypic experiments (Fig. 5B). Initially, CCK-8 assays and cell cycle analysis demonstrated that knockdown of YAP1 significantly inhibited the proliferation of CC cells, while overexpression of YAP1 markedly promoted their proliferative potential (Fig. 5C, D). Flow cytometry experiments indicated that downregulation of YAP1 expression significantly increased the death of CC cells, suggesting that YAP1 may suppress death, allowing cancer cells to evade programmed cell death. Conversely, the overexpression of YAP1 clearly inhibited the apoptotic process, further supporting the notion that YAP1 supports cancer cell survival (Fig. 5D). Additionally, wound healing and Transwell assays provided insights into the impact of YAP1 on the migration and invasion abilities of cervical cancer cells. The results showed that knockdown of YAP1 significantly weakened these abilities, indicating that YAP1 is crucial for the migratory and invasive properties of CC cells. In contrast, overexpression of YAP1 exhibited the opposite effect, significantly enhancing cell migration and invasion (Fig. 5F, G). Subsequent western blot experiments indicated that knocking down YAP1 significantly promoted the expression of ACSL4, whereas overexpressing YAP1 prominently inhibited ACSL4 expression (Fig. 5H, I). Collectively, these findings suggest that the YAP1 gene notably enhances the proliferation, migration, and invasion of cervical cancer cells while also influencing ferroptosis. Mechanistically, YAP1 directly binds to the ACSL4 promoter, enhancing its transcriptional activity and upregulating ACSL4 expression. The conformational stability of YAP1 mediated by PIN1 further regulates this axis, amplifying its transcriptional output. These discoveries highlight the therapeutic potential of targeting the PIN1-YAP1-ACSL4 signaling cascade in cervical cancer treatment.

### Role of YAP1 in ferroptosis in cervical cancer cells

**Fig. 6:**
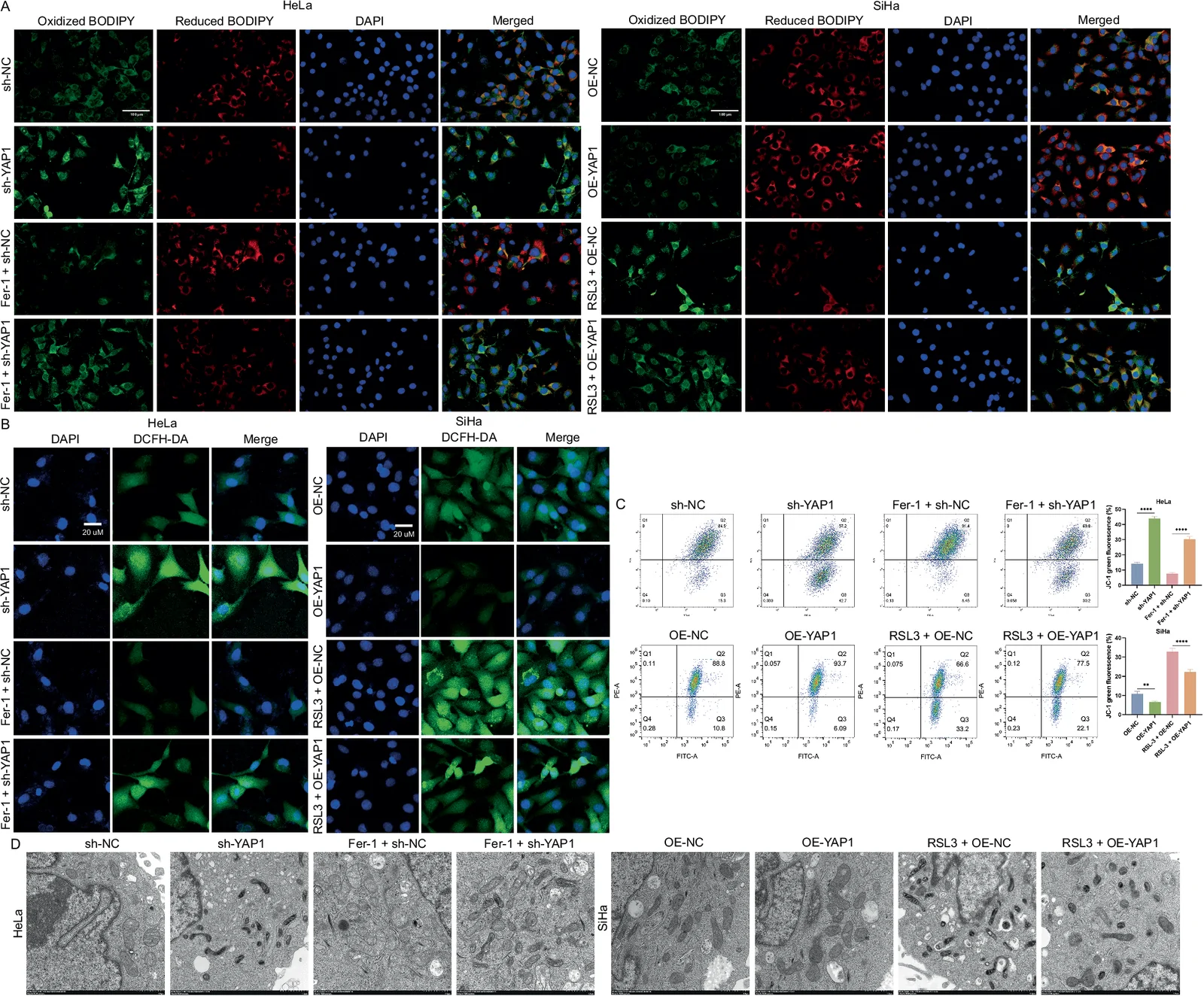
YAP1 inhibits ferroptosis and promotes the progression of cervical cancer. **A** C11-BODIPY 581/591 probe-stained images after YAP1 knockdown and overexpression, as well as after the addition of Fer-1 in the knockdown group and RSL3 in the overexpression group (Bar = 100 μm). **B** ROS immunofluorescence results showing ROS accumulation after YAP1 knockdown and overexpression, as well as after the addition of Fer-1 in the knockdown group and RSL3 in the overexpression group (Bar = 20 μm). **C** JC-1 flow analysis results after YAP1 knockdown and overexpression, as well as after the addition of Fer-1 in the knockdown group and RSL3 in the overexpression group. **D** Electron microscopy observations of mitochondrial structural integrity after YAP1 knockdown and overexpression, as well as after the addition of Fer-1 in the knockdown group and RSL3 in the overexpression group (Bar = 1 μm). **P < 0.01, ****P < 0.0001.

In stable YAP1 knockdown and overexpression cell lines, the addition of ferroptosis inducers and inhibitors yielded significant and insightful findings. Initial lipid ROS detection experiments utilizing C11-BODIPY showed increased green fluorescence in HeLa cells after the knockdown of YAP1, indicating greater lipid ROS production in these cells. Treatment with Fer-1 reduced green fluorescence, effectively reversing the effects of YAP1 silencing. In contrast, overexpression of YAP1 resulted in decreased green fluorescence, while treatment with RSL3 caused increased green fluorescence (Fig. 6A). Staining images obtained using the DCFH-DA probe revealed that knocking down YAP1 significantly enhanced the intensity of ROS fluorescence, indicating a higher level of reactive oxygen species. Conversely, the addition of the ferroptosis inhibitor Fer-1 markedly reduced the ROS fluorescence levels, demonstrating its efficacy in mitigating oxidative stress. Moreover, the suppressive effect of Fer-1 on ROS fluorescence expression was notably and significantly reversed following YAP1 knockdown, highlighting the intricate relationship between YAP1 levels and ferroptosis regulation. In contrast, overexpression of YAP1 resulted in a significant suppression of ROS fluorescence, suggesting that higher YAP1 levels can effectively counteract oxidative stress. However, the introduction of the ferroptosis inducer RSL3 prominently increased ROS fluorescence, indicating that RSL3 can override the protective effects of YAP1 overexpression. Notably, YAP1 overexpression also significantly diminished the enhancing effect of RSL3 on ROS fluorescence, further underscoring the regulatory role of YAP1 in ferroptosis (Fig. 6B). Further analysis using JC-1 flow cytometry provided additional insights into mitochondrial function. The results demonstrated that YAP1 gene knockout led to a significant increase in the percentage of green fluorescence, which is indicative of disrupted mitochondrial membrane potential and compromised mitochondrial health. The addition of Fer-1 notably decreased the percentage of green fluorescence, suggesting that Fer-1 helps in stabilizing the mitochondrial membrane potential. Conversely, YAP1 overexpression caused an increase in red fluorescence, indicating a stabilization and healthier state of the mitochondrial membrane potential. However, with the addition of RSL3, there was again a significant increase in green fluorescence, pointing to compromised mitochondrial function and further highlighting the impact of ferroptosis inducers on mitochondrial integrity (Fig. 6C). Electron microscopy analysis of mitochondrial structures provided a more detailed morphological perspective. Treatment with Fer-1 resulted in mitochondria that appeared more intact and organized, with well-defined cristae, indicative of healthy mitochondrial morphology. In contrast, treatment with RSL3 caused notable mitochondrial shrinkage and cristae loss, suggesting unhealthy and dysfunctional mitochondrial morphology. Interestingly, these effects could be reversed by either knocking down or overexpressing YAP1, demonstrating the pivotal role of YAP1 in maintaining mitochondrial integrity and function (Fig. 6D). In conclusion, these comprehensive results indicate that YAP1 significantly inhibits the progression of ferroptosis in cervical cancer cells by influencing ROS levels and the stability of the mitochondrial membrane potential, thereby weakening the cells’ sensitivity to ferroptosis. This study underscores the crucial role of YAP1 in regulating ferroptosis in cervical cancer cells and suggests that targeting YAP1 could be a potential new therapeutic strategy for the treatment of cervical cancer, offering promising avenues for future research and clinical applications.

### YAP1 suppresses ferroptosis by promoting the autophagic degradation of ACSL4

**Fig. 7:**
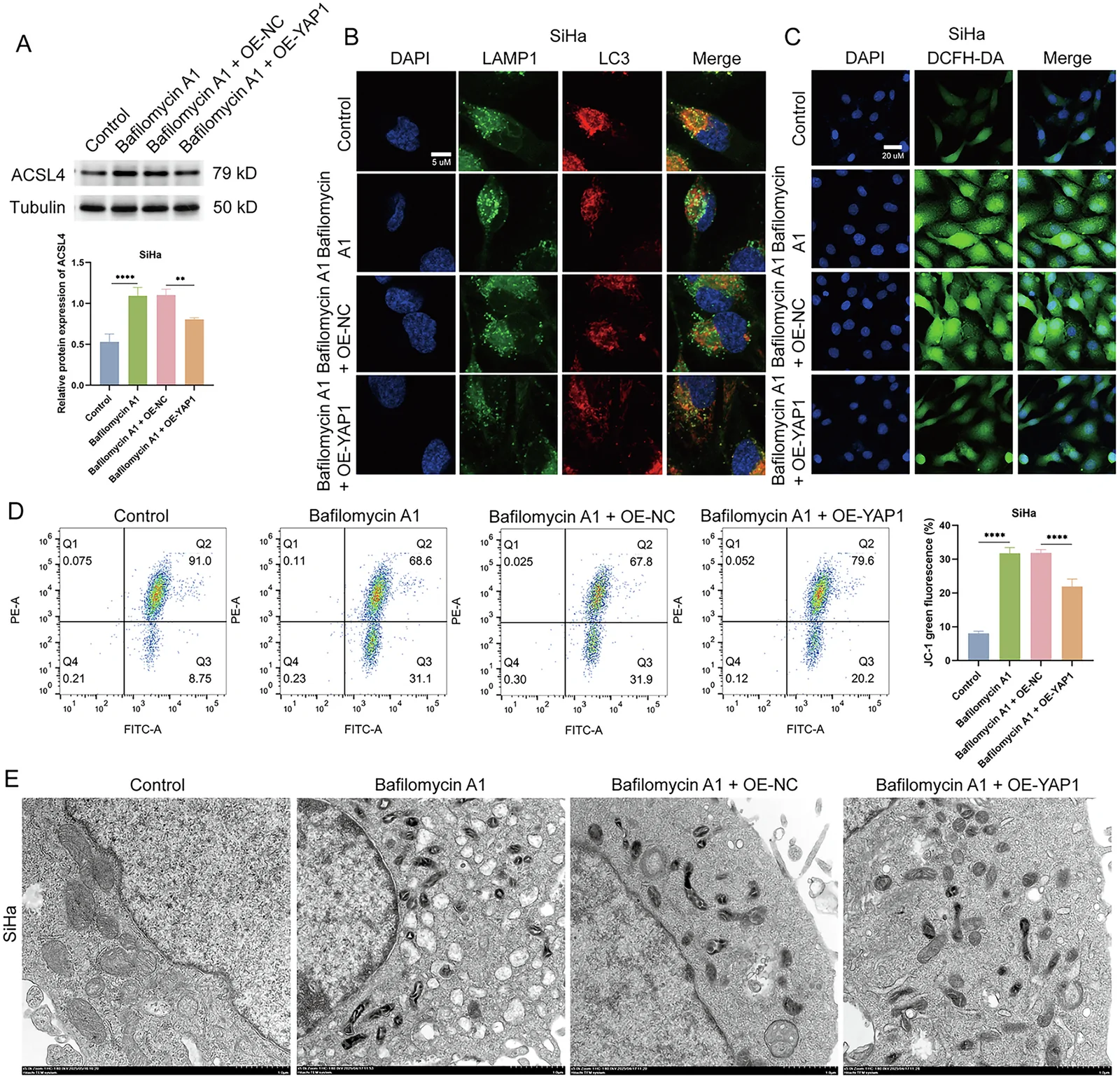
YAP1 suppresses ferroptosis by promoting the autophagic degradation of ACSL4. **A** Western blot validation of the effects of the late-stage autophagy inhibitor Bafilomycin A1 and YAP1 overexpression on ACSL4 protein levels. **B** The effects of the late-stage autophagy inhibitor Bafilomycin A1 and YAP1 overexpression on the fluorescence co-localization of LAMP1 and LC3 (Bar = 5 μm). **C** The effects of the late-stage autophagy inhibitor Bafilomycin A1 and YAP1 overexpression on ROS accumulation (Bar = 20 μm). **D** JC-1 flow analysis results after the addition of the late-stage autophagy inhibitor Bafilomycin A1 and YAP1 overexpression. **E** Electron microscopy observations of mitochondrial structure after the addition of the late-stage autophagy inhibitor Bafilomycin A1 and YAP1 overexpression (Bar = 1 μm). **P < 0.01, ****P < 0.0001.

To delve deeper into understanding how YAP1 inhibits ferroptosis by facilitating the autophagic degradation of ACSL4, we utilized the late-stage autophagy inhibitor Bafilomycin A1 in our experimental setup. Through meticulous WB analyses, we observed that the introduction of Bafilomycin A1 led to a significant upregulation in the expression levels of ACSL4. However, when YAP1 was overexpressed in the system, this upregulation effect induced by Bafilomycin A1 on ACSL4 expression was markedly diminished (Fig. 7A). This suggests that YAP1 plays a crucial role in modulating the autophagic degradation process of ACSL4. Furthermore, our immunofluorescence experiments provided additional insights. The addition of Bafilomycin A1 notably decreased the fluorescence intensity of LAMP1 and LC3, which are key markers of autophagy. Interestingly, the overexpression of YAP1 further attenuated the suppressive effect of Bafilomycin A1 on the fluorescence expression of LAMP1 and LC3 (Fig. 7B). This indicates that YAP1 not only influences the expression levels of ACSL4 but also affects the autophagic machinery at a broader level. In parallel, ROS immunofluorescence assays revealed that Bafilomycin A1 significantly amplified the ROS fluorescence signals, indicative of increased oxidative stress. However, the overexpression of YAP1 notably reduced the enhancing effect of Bafilomycin A1 on ROS fluorescence signals, highlighting the protective role of YAP1 against oxidative damage (Fig. 7C). To assess the impact on mitochondrial function, we employed flow cytometry analysis using the JC-1 dye. The results showed that the addition of Bafilomycin A1 led to a significant increase in the percentage of green fluorescence signals, signifying a disruption in the mitochondrial membrane potential. Conversely, the overexpression of YAP1 decreased the percentage of green fluorescence signals, thereby mitigating the detrimental effects of Bafilomycin A1 on mitochondrial integrity (Fig. 7D). Additionally, electron microscopy observations provided a detailed view of the mitochondrial structural changes. Treatment with Bafilomycin A1 prominently induced mitochondrial shrinkage and a noticeable reduction in the number of cristae, resulting in an incomplete and compromised mitochondrial morphology. In stark contrast, the overexpression of YAP1 preserved the mitochondrial structure, leading to more intact and healthier mitochondrial profiles (Fig. 7D). These comprehensive findings collectively elucidate the intricate molecular network through which YAP1 regulates ferroptosis, particularly by influencing the autophagic degradation of ACSL4. This research not only provides a novel theoretical foundation for developing intervention strategies targeting the YAP1-ACSL4 axis but also underscores the potential therapeutic benefits of modulating YAP1 activity in the context of cancer therapy. By understanding and manipulating this regulatory pathway, we may be able to effectively control ferroptosis, thereby enhancing the efficacy of cancer treatments.

### In vivo experiments to validate the mechanism by which PIN1 regulates ferroptosis in cervical cancer

**Fig. 8:**
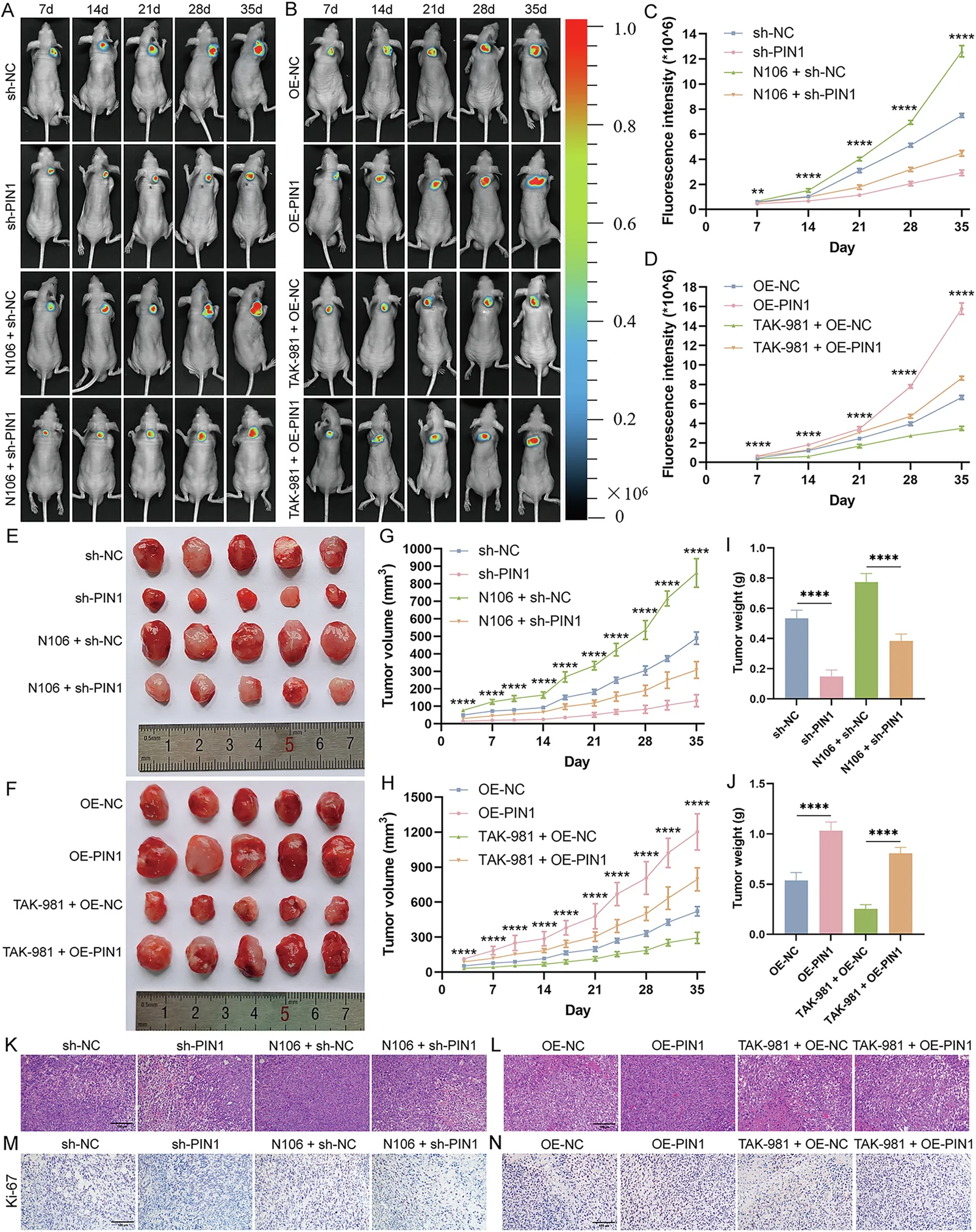
PIN1 promotes the progression of CC in vivo. **A** Morphological presentation of fluorescence intensity after PIN1 knockdown and the addition of the SUMOylation activator N106 on days 7, 14, 21, 28, and 35. **B** Morphological presentation of fluorescence intensity after PIN1 overexpression and the addition of the SUMOylation inhibitor TAK-981 on days 7, 14, 21, 28, and 35. **C** Line graph of fluorescence intensity after PIN1 knockdown and the addition of the SUMOylation activator N106 on days 7, 14, 21, 28, and 35. **D** Line graph of fluorescence intensity after PIN1 overexpression and the addition of the SUMOylation inhibitor TAK-981. **E**, **F** Tumors were harvested from mice on Day 35. **G, H** Tumor volume in each group. **I, J** Final tumor weight in each group. **K** H&E staining results after PIN1 knockdown and the addition of the SUMOylation activator N106 (Bar = 100 μm). **L** H&E staining results after PIN1 overexpression and the addition of the SUMOylation inhibitor TAK-981 (Bar = 100 μm). **M** Ki-67 immunohistochemical results after PIN1 knockdown and the addition of the SUMOylation activator N106 (Bar = 100 μm). **N** Ki-67 immunohistochemical results after PIN1 overexpression and the addition of the SUMOylation inhibitor TAK-981 (Bar = 100 μm). **P < 0.01, ****P < 0.0001.

**Fig. 9:**
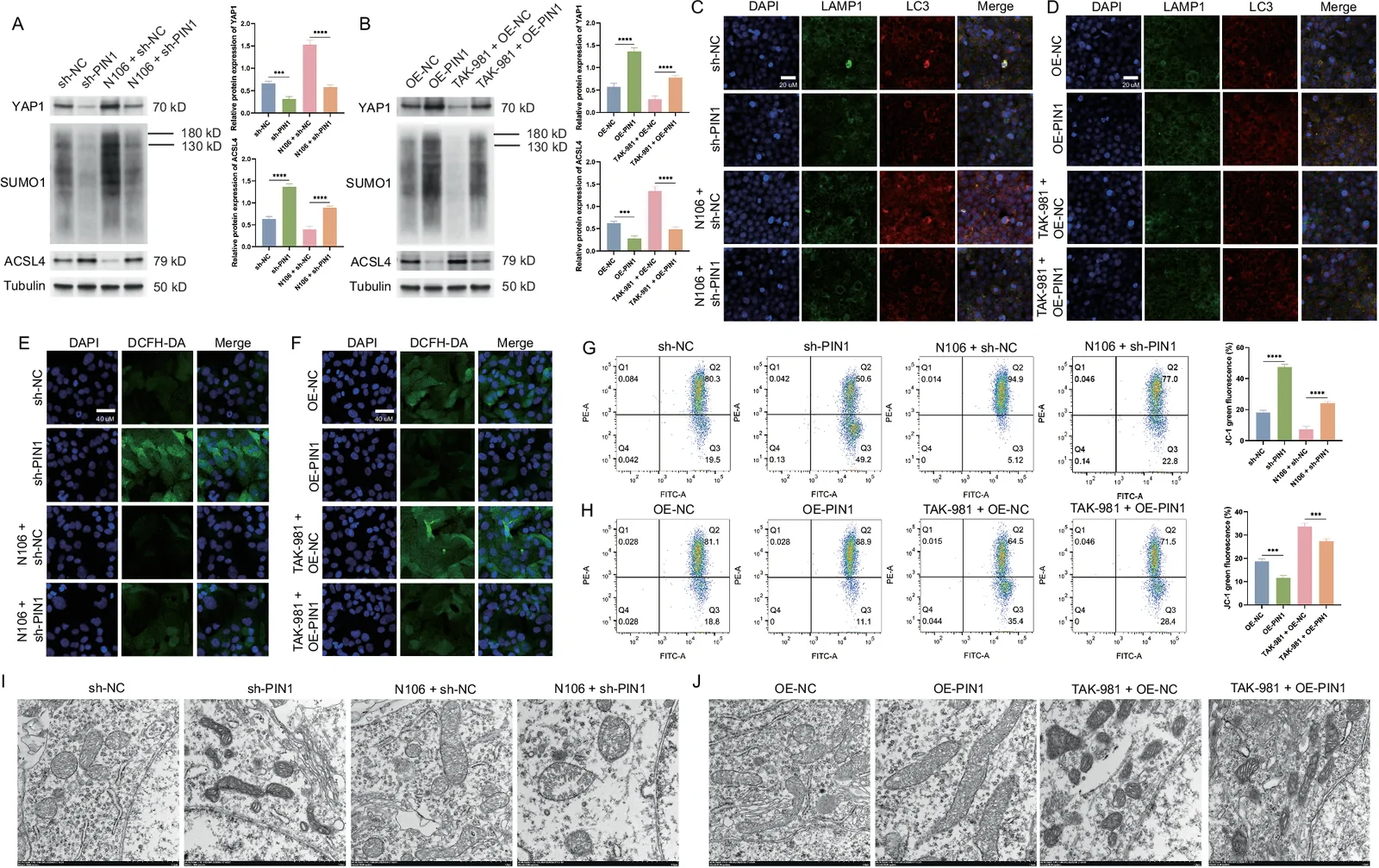
PIN1 regulates the SUMOylation of YAP1 in vivo and promotes the malignant phenotype of CC. **A** Western blot experiments validating the effects of PIN1 knockdown and the addition of the SUMOylation activator N106 on the SUMOylation of YAP1 and the protein levels of ACSL4. **B** Immunoprecipitation experiments validating the effects of PIN1 overexpression and the addition of the SUMOylation inhibitor TAK-981 on the SUMOylation of YAP1 and the protein levels of ACSL4. **C** The effects of PIN1 knockdown and the addition of the SUMOylation activator N106 on the fluorescence co-localization of LAMP1 and LC3 (Bar = 20 μm). **D** The effects of PIN1 overexpression and the addition of the SUMOylation inhibitor TAK-981 on the fluorescence co-localization of LAMP1 and LC3 (Bar = 20 μm). **E** The effects of PIN1 knockdown and the addition of the SUMOylation activator N106 on ROS accumulation (Bar = 40 μm). **F** The effects of PIN1 overexpression and the addition of the SUMOylation inhibitor TAK-981 on ROS accumulation (Bar = 40 μm). **G** JC-1 flow analysis results after PIN1 knockdown and the addition of the SUMOylation activator N106. **H** JC-1 flow analysis results after PIN1 overexpression and the addition of the SUMOylation inhibitor TAK-981. **I** Electron microscopy observations of mitochondrial structure after PIN1 knockdown and the addition of the SUMOylation activator N106 (Bar = 1 μm). **J** Electron microscopy observations of mitochondrial structure after PIN1 overexpression and the addition of the SUMOylation inhibitor TAK-981 (Bar = 1 μm). ***P < 0.001, ****P < 0.0001.

Using PIN1 knockout and overexpression cervical cancer xenograft models, we further validated the critical role of PIN1 in regulating ferroptosis. In our experiments, we detected fluorescence intensity at different time points (7, 14, 21, 28, and 35 days) and found that knockdown of PIN1 significantly inhibited the intensity of the fluorescence signal, while overexpression of PIN1 significantly enhanced the intensity of the fluorescence signal, and these could be reversed by N106 or TAK-981 (Fig. 8A–D). These results were also verified in the detection of tumor volume and weight (Fig. 8E–J). The HE staining results indicated that after PIN1 knockdown, the tissue morphology was more intact, and the cellular arrangement was more compact. In contrast, in the PIN1 overexpression group, more severe cellular infiltration was observed, and the arrangement of cells was noticeably disordered (Fig. 8K, L). Immunohistochemical results showed that knockdown of PIN1 significantly reduced the level of Ki-67 infiltration, while N106 promoted the expression of Ki-67, which was suppressed by the knockdown of PIN1. Overexpression of PIN1 significantly enhanced Ki-67 infiltration. However, the addition of TAK-981 resulted in a notable decrease in Ki-67 expression, an effect that was reversed by overexpression of PIN1 (Fig. 8M, N). Furthermore, we assessed the SUMOylation levels in mouse tissues, revealing that PIN1 knockdown significantly inhibited the expression levels of YAP1 and SUMO1 while significantly promoting the expression of ACSL4. The addition of N106 markedly enhanced the expression levels of YAP1 and SUMO1 and significantly inhibited the expression of ACSL4, while PIN1 knockdown appeared to significantly weaken the phenotypic effects of N106. Overexpression of PIN1 resulted in a significant increase in YAP1 and SUMO1 levels, along with suppression of ACSL4 expression. After the addition of TAK-981, YAP1 expression and SUMO1 levels significantly decreased, but overexpression of PIN1 could notably reverse the effects of TAK-981 (Fig. 9A, B). Additionally, we evaluated the colocalization abilities of autophagy and lysosomal-related markers under the influence of SUMOylation inhibitors and activators. Immunofluorescence results showed that knockdown of PIN1 significantly reduced the fluorescence signals of LAMP1 and LC3, while the addition of N106 dramatically enhanced the colocalization with LAMP1 and LC3; conversely, PIN1 overexpression and the addition of TAK-981 exhibited opposite effects (Fig. 9C, D). For the immunofluorescence detection of ROS, we found that knockdown of PIN1 significantly increased ROS fluorescence signals, while the addition of N106 notably decreased ROS levels. TAK-981, on the other hand, significantly raised ROS levels (Fig. 9E, F). Subsequent analysis via JC-1 flow cytometry indicated that PIN1 gene knockout led to a significant increase in the proportion of green fluorescence, suggesting disruption of mitochondrial membrane potential; however, the addition of N106 resulted in a significant increase in red fluorescence percentage. Overexpression of PIN1 was associated with increased red fluorescence, indicating stable maintenance of mitochondrial membrane potential, whereas the addition of TAK-981 significantly increased the percentage of green fluorescence (Fig. 9G, H). Further analysis of mitochondrial structure using electron microscopy indicated that treatment with N106 resulted in more intact mitochondria with organized cristae, while treatment with TAK-981 led to significant mitochondrial shrinkage and cristae detachment, indicative of unhealthy mitochondrial morphology (Fig. 9I, J). These findings further reinforce the important role of PIN1 in the regulation of ferroptosis and provide a potential theoretical basis for intervention targeting the PIN1-ACSL4 axis, which has significant implications for the development of new therapeutic strategies.

### In vivo experiments to validate the mechanism by which YAP1 regulates ferroptosis in cervical cancer

**Fig. 10:**
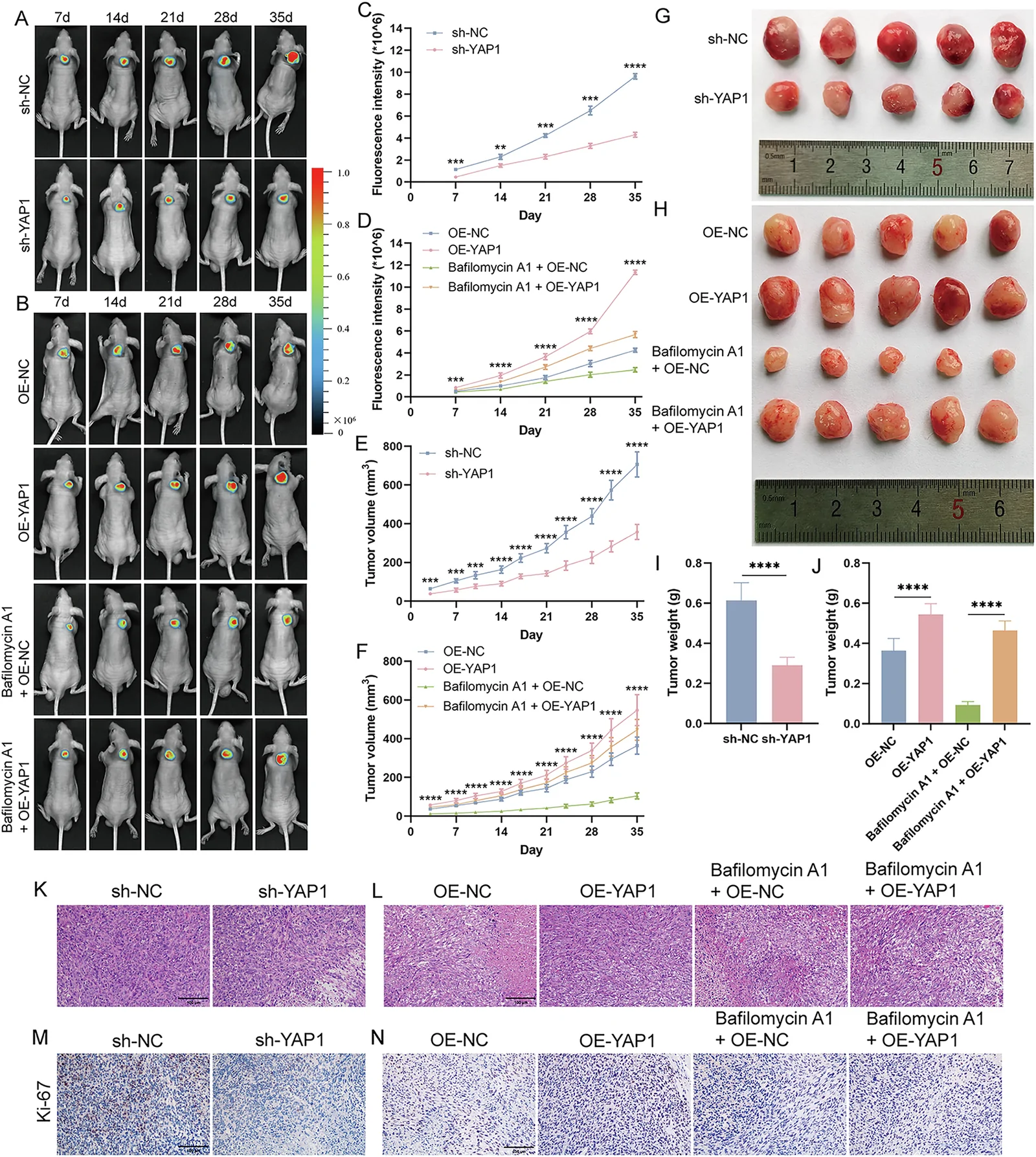
YAP1 promotes the progression of CC in vivo. **A** Morphological presentation of fluorescence intensity after YAP1 knockdown on days 7, 14, 21, 28, and 35. **B** Morphological presentation of fluorescence intensity after YAP1 overexpression and the addition of the late-stage autophagy inhibitor Bafilomycin A1 on days 7, 14, 21, 28, and 35. **C** Line graph of fluorescence intensity after YAP1 knockdown on days 7, 14, 21, 28, and 35. **D** Line graph of fluorescence intensity after YAP1 overexpression and the addition of the late-stage autophagy inhibitor Bafilomycin A1. **E, F** Tumor volume in each group. **G, H** Tumors were harvested from mice on Day 35. **I, J** Final tumor weight in each group. **K** H&E staining results after YAP1 knockdown (Bar = 100 μm). **L** H&E staining results after YAP1 overexpression and the addition of the late-stage autophagy inhibitor Bafilomycin A1 (Bar = 100 μm). **M** Ki-67 immunohistochemical results after YAP1 knockdown (Bar = 100 μm). **N** Ki-67 immunohistochemical results after YAP1 overexpression and the addition of the late-stage autophagy inhibitor Bafilomycin A1 (Bar = 100 μm). **P < 0.01, ***P < 0.001, ****P < 0.0001.

**Fig. 11:**
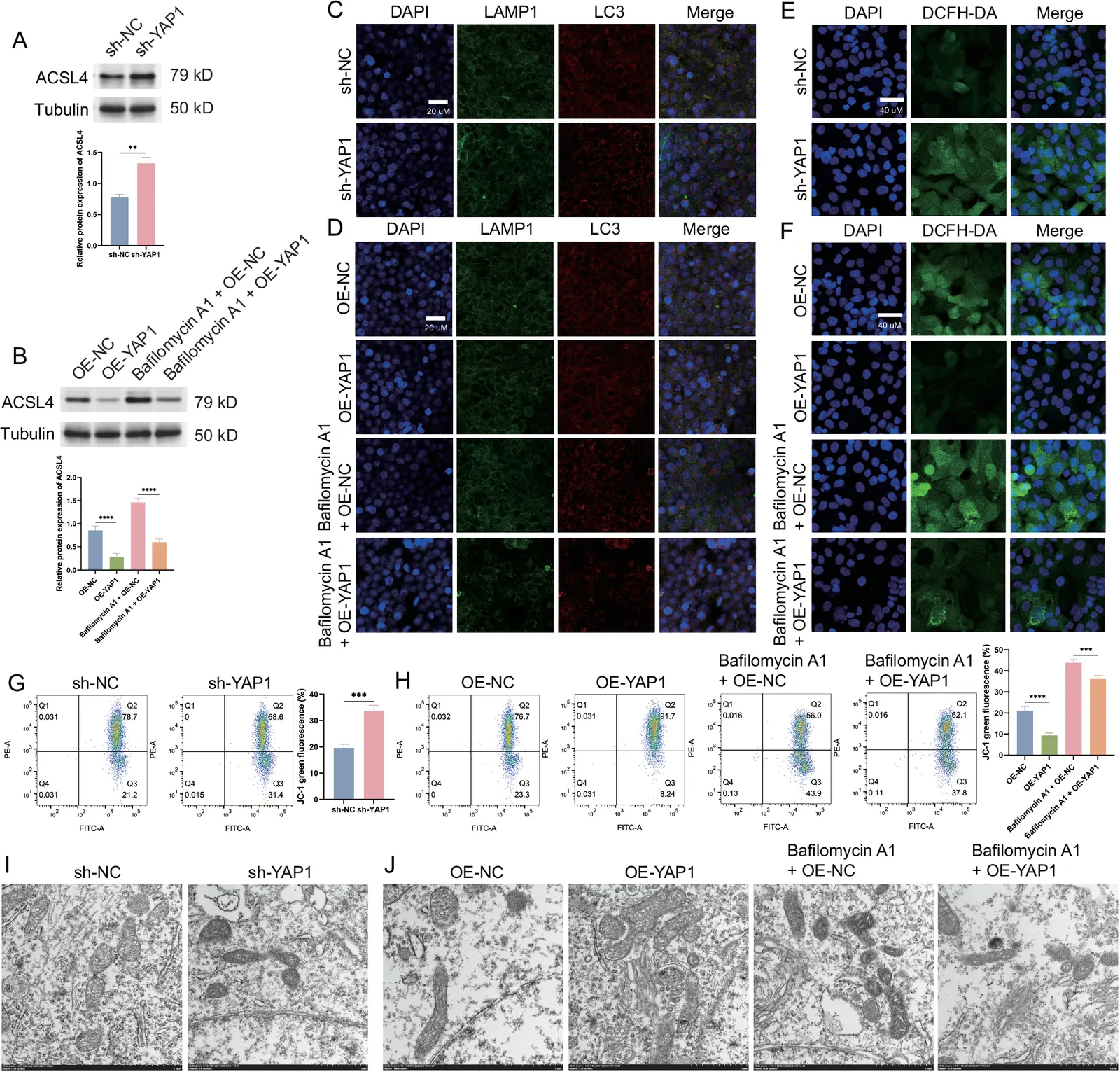
YAP1 regulates the autophagy level of ACSL4 in vivo and promotes the malignant phenotype of CC. **A** Western blot experiment validating the effects of YAP1 knockdown on ACSL4 protein levels. **B** Western blot experiments validating the effects of YAP1 overexpression and the addition of the late-stage autophagy inhibitor Bafilomycin A1 on the SUMOylation of YAP1 and ACSL4 protein levels. **C** The effects of YAP1 knockdown on the fluorescence co-localization of LAMP1 and LC3 (Bar = 20 μm). **D** The effects of YAP1 overexpression and the addition of the late-stage autophagy inhibitor Bafilomycin A1 on the fluorescence co-localization of LAMP1 and LC3 (Bar = 20 μm). **E** The effects of YAP1 knockdown on ROS accumulation (Bar = 40 μm). **F** The effects of YAP1 overexpression and the addition of the late-stage autophagy inhibitor Bafilomycin A1 on ROS accumulation (Bar = 40 μm). **G** JC-1 flow analysis results after YAP1 knockdown. **H** JC-1 flow analysis results after YAP1 overexpression and the addition of the late-stage autophagy inhibitor Bafilomycin A1. **I** Electron microscopy observations of mitochondrial structure after YAP1 knockdown (Bar = 1 μm). **J** Electron microscopy observations of mitochondrial structure after YAP1 overexpression and the addition of the late-stage autophagy inhibitor Bafilomycin A1 (Bar = 1 μm). **P < 0.01, ***P < 0.001, ****P < 0.0001.

Utilizing YAP1 knockout and overexpression cervical cancer xenograft models, we further elucidated the critical role of YAP1 in the regulation of ferroptosis. Our experimental results demonstrated a time-dependent decrease in fluorescence intensity at various intervals (7, 14, 21, 28, and 35 days) following YAP1 knockdown, indicating a significant inhibition of the fluorescence signal. Conversely, overexpression of PIN1 markedly enhanced the fluorescence intensity. The introduction of Bafilomycin A1 led to a notable reduction in fluorescence signal intensity compared to the YAP1 overexpression group; however, this effect was reversed by YAP1 overexpression (Fig. 10A–D). These results were also verified in the detection of tumor volume and weight (Fig. 10E–J). Histological analysis through HE staining revealed that YAP1 knockdown resulted in more intact tissue morphology with a more compact cellular arrangement. In contrast, the YAP1 overexpression cohort exhibited pronounced cellular infiltration and significant disorder in cell arrangement. Although the addition of Bafilomycin A1 resulted in a reduction in the extent of malignant infiltration, YAP1 overexpression exacerbated such infiltration (Fig. 10K, L). Immunohistochemical analysis indicated that YAP1 knockdown significantly decreased Ki-67 expression, a marker associated with cellular proliferation. In stark contrast, YAP1 overexpression led to a significant increase in Ki-67 expression, which was notably attenuated with the addition of Bafilomycin A1, though this effect was negated by YAP1 overexpression (Fig. 10M, N). Furthermore, in tumour tissues, YAP1 knockdown markedly elevated ACSL4 expression, whereas YAP1 overexpression substantially inhibited it. Bafilomycin A1 treatment significantly increased ACSL4 expression, an effect mitigated by YAP1 overexpression (Fig. 11A, B). We also investigated the colocalization of autophagy and lysosomal markers in the context of Bafilomycin A1 administration. Immunofluorescence analysis revealed that YAP1 knockdown significantly diminished the fluorescence signals of LAMP1 and LC3. Conversely, YAP1 overexpression significantly enhanced the fluorescence of LAMP1 and LC3, and the introduction of Bafilomycin A1 substantially decreased their colocalization; however, this inhibition was significantly diminished by YAP1 overexpression (Fig. 11C, D). The assessment of ROS using immunofluorescence indicated that YAP1 knockdown significantly increased ROS fluorescence intensity. In contrast, YAP1 overexpression led to a notable decrease in ROS fluorescence signals, which dramatically increased upon Bafilomycin A1 treatment, although this increase was further attenuated by YAP1 overexpression (Fig. 11E, F). Flow cytometry analysis using JC-1 confirmed that YAP1 knockdown resulted in a significant increase in the proportion of green fluorescence, indicative of disrupted mitochondrial membrane potential. In contrast, the administration of Bafilomycin A1 led to a substantial increase in green fluorescence percentage, and YAP1 overexpression was associated with enhanced red fluorescence (Fig. 11G, H). Ultrastructural analysis of mitochondrial morphology via electron microscopy showed that YAP1 knockdown resulted in atrophied mitochondria with reduced cristae. Conversely, YAP1 overexpression led to well-preserved and organized mitochondrial structures. Post-treatment with Bafilomycin A1 revealed significant mitochondrial shrinkage and cristae detachment, indicative of compromised mitochondrial health (Fig. 11I, J). Collectively, these findings underscore the pivotal role of YAP1 in ferroptosis regulation and provide a theoretical framework for targeting the PIN1-YAP1-ACSL4 signaling axis, which holds significant promise for the development of novel therapeutic strategies in cervical cancer.

## Discussion

Our study elucidates a previously unrecognized function of PIN1 in modulating ferroptotic sensitivity through mitochondrial integrity maintenance. The PIN1-YAP1-ACSL4 axis emerges as a critical regulator of redox homeostasis and lipid peroxidation, key determinants of ferroptosis execution. Notably, the interplay between YAP1 and mitochondrial ultrastructure provides mechanistic insight into how cancer cells resist oxidative damage. These results align with recent evidence linking Hippo pathway effectors to metabolic resilience. Targeting this axis may sensitize cervical cancer cells to ferroptosis-inducing therapies, offering a synergistic approach in treating resistant malignancies.

PIN1, as an oncogene, has been reported to be involved in the development of various cancers[28–31]. Research by Liao and colleagues found that USP34 can upregulate PIN1 expression and SUMOylation, which inhibits ferroptosis by suppressing the cGAS-STING pathway, thereby promoting cervical cancer progression [32]. Additionally, Zhang and colleagues confirmed that PIN1 affects ferroptosis and sensitivity to cisplatin (DDP) in cervical cancer cells through the NRF2/GPX4 axis, identifying PIN1 as a potential therapeutic target for cervical cancer [33]. Furthermore, the study by Ma and others indicated that AMF induces ferroptosis by inhibiting PIN1, thereby suppressing the proliferation, migration, invasion, and inflammation of rheumatoid arthritis fibroblast-like synoviocytes (RA-FLS) [34]. Thus, PIN1 plays a crucial role in regulating ferroptosis and exacerbating tumor progression. Moreover, within oncogenic pathways, the Hippo signaling cascade plays a central role in controlling organ size, tissue homeostasis, and cell fate. Its core effectors, Yes-associated protein (YAP) and transcriptional co-activators with PDZ-binding motifs (TAZ), are key transcriptional co-activators [35–38]. In recent years, abnormal activation of the Hippo signaling pathway has been widely reported in various tumors, with YAP being a critical effector implicated in the regulation of cell proliferation, survival, and sensitivity to ferroptosis [39–42]. Recent studies have indicated that post-translational modifications, particularly SUMOylation, play a vital regulatory role in YAP activity [43–45]. Research shows that YAP1 can promote the progression of cervical cancer through various pathways, and findings indicate that Hippo pathway transcription factors YAP and TAZ play HPV-type-dependent roles in cervical cancer [46]. CircMAST1 competitively sequesters NAT10 and obstructs ac4C modification of YAP mRNA, promoting its degradation and inhibiting tumor progression in cervical cancer [47]. The Ma team’s study identified the WDR5/YAP1/CTGF pathway as a key mechanism in cervical cancer progression [48]. Therefore, YAP1 is a critical molecule in regulating the malignant progression of cervical cancer. Here, we indicated that PIN1 is a novel regulatory factor that enhances YAP1 SUMOylation, thereby suppressing its transcriptional activity and the expression of downstream pro-ferroptosis genes. Mechanistically, PIN1 interacts with YAP1, facilitating its SUMO conjugation and subsequent nuclear exclusion. It directs ACSL4 to autophagic bodies and mediates its degradation through selective autophagy pathways, significantly reducing the cells’ sensitivity to ferroptosis. This process not only unveils a new mechanism for the regulation of YAP1 protein homeostasis but also presents potential therapeutic targets for intervening in ferroptosis-related diseases. In cervical cancer cell models, overexpression of PIN1 significantly enhances YAP1 expression and ACSL4 degradation, suppressing ACSL4-mediated lipid peroxide accumulation and endowing cells with stronger resistance to ferroptosis. The discovery of this regulatory axis expands the molecular connectivity network between the Hippo pathway and cell fate determination. Under stress conditions, the dynamic balance of the PIN1-YAP1 axis becomes a key switch determining cell survival versus death. This regulatory mechanism exhibits significant functionality in tumor resistance, particularly playing a crucial role in chemotherapy-induced ferroptosis resistance. This finding provides a new strategy for reversing the ferroptosis resistance of tumor cells. Targeting the PIN1-YAP1 interaction may enhance the efficacy of conventional chemotherapy drugs, especially in malignant tumors with high PIN1 expression, such as cervical cancer, showing clinical translational potential.

Of course, this study also has some limitations. First, our identification of the PIN1 gene relies on analyses conducted using public databases, and we did not perform data mining in experimental samples. Second, we did not conduct analyses and validations in clinical samples. Third, our research on the SUMOylation of PIN1 and YAP1 is not yet comprehensive. However, these limitations will be the focus of our future work, and we will carry out subsequent research addressing the issues mentioned above.

## Conclusion

In summary, our findings elucidate a novel regulatory axis involving PIN1-mediated YAP1 activation that drives ferroptosis resistance in CC. By integrating bioinformatics prediction with functional validation, we demonstrate that disrupting the PIN1-YAP1 interaction sensitizes CC cells to ferroptosis inducers and chemotherapeutic agents. Although further mechanistic exploration and clinical validation are needed, this study highlights a promising therapeutic strategy for overcoming treatment resistance in high-PIN1-expressing CC.

## Materials and methods

### Cell culture

Cells were cultured in Dulbecco’s Modified Eagle Medium (DMEM) supplemented with 10% fetal bovine serum and 1% penicillin-streptomycin at 37 °C under 5% CO_2_. Human cervical cancer cell lines HeLa (CL-0101) and SiHa (CL-0210) were obtained from Procell Life Science & Technology Co., Ltd. (Wuhan, China). Cells were routinely tested for mycoplasma contamination and authenticated by short tandem repeat profiling. For experimental treatments, cells were seeded at appropriate densities and allowed to adhere overnight before transfection or drug administration. All culture reagents were purchased from Gibco and used as per the manufacturer’s instructions.

### Plasmids, RNA interference, and construction of stable cell lines

The pLKO.1-shPIN1 and pLKO.1-shNC plasmids were procured from Addgene (Beijing, China), whereas the pcDNA3.1-PIN1 was constructed through the cloning of the full-length PIN1 open reading frame (ORF) into the vector. A lentivirus-based short hairpin RNA (shRNA) approach was adopted, employing three distinct shRNA sequences to downregulate the expression of PIN1 and YAP1 in HeLa cells. These shRNA sequences were cloned into the lentiviral vector pLKO.1. Concurrently, the full-length PIN1 and YAP1 complementary DNA (cDNA) were synthesized via quantitative reverse transcription polymerase chain reaction (qRT-PCR) and cloned into the lentiviral overexpression vector pLV-CMV-MCS-PGK-Puro to induce PIN1 overexpression in SiHa cells. For lentivirus production, 10 µg of pLV-CMV-MCS-PGK-Puro was co-transfected with 7.5 µg of the packaging plasmid (pMDLg/pRRE), 2.5 µg of the envelope plasmid (VSV-G), and 15 µg of the helper plasmid (pRSV-Rev) using the calcium phosphate transfection method as part of a third-generation lentiviral system. The transfection was carried out at 37 °C for 48 h. Lentiviral particles were generated by co-transfecting 293 T cells (Procell Life Science & Technology Co., Ltd.) with a packaging plasmid mixture (REV: VSVG: PMDL = 2:3:5) using the TurboFect transfection reagent (R0532, Fermentas, Thermo Fisher Scientific, Inc.). The virus supernatant was harvested 36–48 h post-transfection and filtered for use in infection. HeLa and SiHa cells were seeded in 6-well plates until they reached 60–70% confluency. Subsequently, they were infected with lentiviral particles at a multiplicity of infection (MOI) of 10, supplemented with poly-L-lysine (8 μg/ml) to enhance transduction. After 12–16 h of co-culture with the virus, the medium was replaced with fresh culture medium. After 48 h, stable transductants were selected using 2 µg/ml puromycin (the original concentration is 1 mg/ml) to confirm successful transduction. All procedures were conducted at 37 °C with 5% CO₂. The sequences employed for PIN1 and YAP1 knockdown and overexpression are presented in Table S1.

### RNA extraction and qRT-PCR

Total RNA was extracted using TRIzol reagent according to the manufacturer’s protocol. Equal amounts of RNA were reverse transcribed into cDNA using a PrimeScript RT reagent kit. Quantitative PCR was performed on a CFX96 Real-Time PCR Detection System with SYBR Green master mix. GAPDH was used as an internal control. The relative expression levels were calculated using the 2^⁻ΔΔCT^ method. All primers are listed in Table S2. Reactions were run in triplicate, and experiments were repeated three times independently to ensure reproducibility.

### Cell counting kit-8 (CCK-8) assay

Cells were seeded in 96-well plates at a density of 3 × 10³ cells per well and cultured for 24, 48, 72, or 96 h. At each time point, 10 μL of CCK-8 reagent was added to each well and incubated for 2 h at 37 °C. Absorbance was measured at 450 nm using a microplate reader.

### Flow cytometry cell cycle assay

Cells were harvested, washed with PBS, and fixed in 70% ethanol at 4 °C overnight. Subsequently, the fixed cells were centrifuged and resuspended in PBS containing 1% bovine serum albumin and 0.1% sodium azide. After washing, cells were incubated with RNase A at 37 °C for 30 min, then stained with propidium iodide in the dark at 4 °C for 30 min. Cell cycle distribution was analyzed using a flow cytometer, with data processed by ModFit software to determine the proportions of cells in G0/G1, S, and G2/M phases, reflecting cellular proliferation status.

### Flow cytometry cell death assay

To evaluate cell death, an Annexin V/PI staining assay was performed. Cells were seeded in 6-well plates and exposed to specific experimental conditions. After treatment, the cells were harvested by trypsinization, rinsed twice with cold PBS, and then resuspended in binding buffer. Subsequently, 1 × 10⁶ cells were incubated with 5 μL of Annexin V-FITC and 5 μL of PI solution for 15 min at room temperature in the dark. Following incubation, 400 μL of binding buffer was added to each sample, and the cells were promptly analyzed by flow cytometry (BD FACSCanto).

### Wound healing assay

Cells were inoculated into 6-well plates and cultured until they reached 90% confluence. A wound was generated by scraping the cell monolayer with a 200 μL pipette tip, and then the monolayer was rinsed with phosphate-buffered saline (PBS) to eliminate cellular debris. Microscopic images were acquired at 0, 24, and 48 h post-scraping. The migration distance was quantified utilizing ImageJ software, and the relative wound closure rate was computed as a percentage of the initial gap width.

### Transwell assay

In serum-free medium, 1×10^5^ cells were placed into the upper part of a Transwell insert. Meanwhile, the lower chamber was added with medium that contained 10% fetal bovine serum acting as a chemoattractant. Following 24-h incubation at 37 °C, non-migrated cells on the upper side were wiped off using a cotton swab. Migrated cells on the lower membrane side were then fixed with 4% paraformaldehyde, dyed with 0.1% crystal violet, and observed under a microscope. For each insert, cell counts were carried out in six random fields to determine the average migration or invasion ability.

### Detection of lipid peroxidation

Lipid peroxidation levels were assessed using the C11-BODIPY 581/591 kit (Invitrogen, USA). HeLa and SiHa cells were seeded in 6-well plates and cultured to approximately 5 × 10⁴ cells per well. Following treatment, cells were incubated with 5 μM C11-BODIPY probe (λex = 581 nm, λem = 591 nm) at 37 °C for 30 min according to the manufacturer’s instructions. Fluorescence imaging was performed using a fluorescence microscope (Zeiss, Germany). Images were acquired using conventional Texas Red (581/591 nm) and FITC (488/510 nm) filter sets. Oxidized BODIPY was detected at the excitation and emission maxima of 488 nm and 510 nm, respectively.

### ROS immunofluorescence detection

Cells were seeded onto sterile glass coverslips placed in 6-well plates and allowed to adhere overnight under standard culture conditions. The next day, cells were washed with PBS and incubated with 10 μM DCFH-DA probe at 37 °C for 30 min in the dark. After incubation, the cells were washed three times with PBS to remove excess probe and then immediately visualized under a fluorescence microscope using excitation at 488 nm and emission at 525 nm. Green fluorescence intensity was proportional to intracellular ROS levels. All procedures were performed under minimal light exposure to prevent probe degradation, and samples were analyzed promptly to ensure accurate detection.

### Mitochondrial membrane potential JC-1

HeLa and SiHa cells were seeded in 6-well plates at a density ranging from 1 × 10⁵ to 2 × 10⁵ cells per well and incubated for 24 h at 37 °C in an incubator with a 5% CO₂ atmosphere to facilitate cell adhesion and proliferation. After this incubation period, the cells were subjected to specific experimental treatments such as adding ferroptosis inhibitor Fer-1 (MCE, HY-100579) and inducer RSL3 (MCE, HY-100218A), SUMOylation inhibitor TAK-981 (MCE, HY-111789), SUMOylation activator N106 (MCE, HY-110273), and late-stage autophagy inhibitor Bafilomycin A1 (MCE, HY-100558), and gently rinsed with PBS. Subsequently, the cells were detached using trypsin/EDTA solution at 37 °C for 5 to 10 min. The resulting cell suspension was then collected and centrifuged at 300–400 × *g* for 5 min. After discarding the supernatant, the cell pellet was resuspended in 1 mL of fresh culture medium or PBS. An aliquot containing 0.5 to 1 × 10⁶ cells was transferred to a new tube, and JC-1 dye was added to the cell suspension in accordance with the manufacturer’s protocol. The cells were then incubated in the dark at 37 °C for 20 to 30 min. Immediately after incubation, the cells were analyzed by flow cytometry. The flow cytometer settings were adjusted to detect the red fluorescence emitted by J-aggregates, which is indicative of a high mitochondrial membrane potential, and the green fluorescence of JC-1 monomers, which is indicative of a low mitochondrial membrane potential. The appropriate fluorescence detection channels were employed, specifically 585 nm for red fluorescence and 530 nm for green fluorescence.

### Mitochondrial structure detecting

The specimens were fixed in 2.5% glutaraldehyde (prepared with phosphate-buffered saline) for a duration of 2 h, after which they were washed with 0.1 M phosphate-buffered saline. Subsequently, the specimens were fixed in a 1% osmium tetroxide solution for 2 h and washed once again with 0.1 M phosphate-buffered saline. Thereafter, dehydration was carried out using graded ethanol at 4 °C. The specimens were treated with a mixture of pure acetone and embedding medium at a ratio of 2:1 at room temperature for 4 h, followed by treatment with a mixture of pure acetone and embedding medium at a ratio of 1:2 overnight. Subsequently, the specimens were incubated in pure embedding medium at 37 °C for 3 h. Ultrathin sections, with a thickness of approximately 50 nm, were obtained using an ultramicrotome. The sections were subjected to double-staining with 3% uranyl acetate and lead citrate and examined via transmission electron microscopy.

### Western blot

Western blot analysis was performed to detect protein expression levels. Cells were lysed in RIPA buffer supplemented with protease and phosphatase inhibitors, and protein concentrations were determined using a bicinchoninic acid (BCA) assay. Equal amounts of protein were separated by SDS-PAGE and transferred to PVDF membranes. Membranes were blocked with 5% non-fat milk and incubated with primary antibodies against PIN1 (Proteintech, 10495-1-AP, 1:1000), YAP1 (ABclonal, A19134, 1:10000), SUMO1 (Cell Signaling Technology, 4930S, 1:1000), SUMO2/3 (Cell Signaling Technology, 4971S, 1:1000), ACSL4 (ABclonal, A20414, 1:10000), and Tubulin (ABclonal, AC015, 1:1000) at 4 °C overnight, followed by HRP-conjugated secondary antibodies. The blots were visualized using the ECL chemiluminescence system (Bioground), and densitometric analysis was conducted with e-BLOT software. Tubulin was employed as the internal control.

### Co-immunoprecipitation (Co-IP)

Co-IP assays were performed to investigate the interactions between target proteins in HeLa and SiHa cells treated with the corresponding lentivirus and reagents. Cell lysates were incubated with a primary antibody against the target protein overnight at 4 °C, followed by the addition of protein A/G magnetic beads for 2 h. After washing, bound proteins were eluted and subjected to SDS-PAGE and Western blot analysis to detect interacting partners, confirming specific protein-protein interactions under physiological conditions.

### Immunofluorescence assay

Cells grown on coverslips were fixed with 4% paraformaldehyde, permeabilized with 0.1% Triton X-100, and blocked with 5% BSA. Primary antibodies were incubated overnight at 4 °C, followed by Alexa Fluor-conjugated secondary antibodies and DAPI for nuclear staining. Primary antibodies included those against PIN1 (Proteintech, 10495-1-AP, 1:200), YAP1 (ABclonal, A19134, 1:200), LAMP1 (Proteintech, 67300-1-Ig, 1:200), and LC3 (Proteintech, 14600-1-AP, 1:250). Fluorescence signals were visualized using a confocal laser scanning microscope, revealing precise spatial distribution patterns consistent with functional implications in cellular processes.

### Construction of a xenograft tumor model

Male nude BALB/c mice (4–6 weeks old, weighing 18–22 grams) were kept in an environment of 20–24 °C with a humidity of 50–60%, under a light/dark cycle of 12/12 h, with free access to food and water. HeLa and SiHa cells transfected with either an empty vector or the PIN1 and YAP1 shRNA or overexpression vector (4.0 × 10^7^ cells/mL) were suspended in PBS and subcutaneously injected into the dorsal neck of the nude mice (*n* = 5). On days 7, 14, 21, 28, and 35 post-injection, tumor size was estimated based on bioluminescence measurements. The volume of the tumor was measured using the caliper twice a week, and the weight of the tumor was measured on day 35 post-implantation. On day 35, the mice were euthanized by cervical dislocation after being anesthetized with 2% sodium pentobarbital (50 mg/kg). In the process of euthanizing the mice, we first administered a sedative (5 mg/kg) via intraperitoneal injection. Once the animals were tranquilized and unconscious, we proceeded with an intravenous injection of an overdose of sodium pentobarbital (150 mg/kg). We then monitored the mice’s heart rate, respiration, and pupillary reflexes to confirm their death within a short period. Subsequently, all tumor tissues from the mice were collected and weighed. This study was approved by the Institutional Animal Care and Use Committee of the Hubei Provincial Center for Disease Control and Prevention.

### Hematoxylin and eosin (HE) staining

HE staining was employed to assess the histological characteristics of tissues. Normal and tumor tissues were fixed in 10% formalin, embedded in paraffin blocks, and sectioned into slices with a thickness of 5 microns, which were placed on microscope slides. After deparaffinization and rehydration, the sections were stained with hematoxylin to make the cell nuclei visible, followed by counterstaining with eosin to emphasize the cytoplasm. Subsequently, the stained sections were observed under a microscope, and images were collected for subsequent analysis.

### Immunohistochemical (IHC) testing

IHC testing was performed to evaluate the expression levels of specific biomarkers in tissues. Paraffin-embedded tissue sections were deparaffinized, rehydrated, and subjected to antigen retrieval. Endogenous peroxidase activity was blocked with 3% hydrogen peroxide, followed by incubation with primary antibodies against Ki-67 (27309-1-AP, 1:4000, Proteintech) at 4 °C overnight. Sections were then incubated with secondary antibodies conjugated to horseradish peroxidase, and signals were visualized using diaminobenzidine substrate. Nuclei were counterstained with hematoxylin, and slides were examined under a light microscope to assess staining intensity and distribution. Positive staining was scored based on intensity and the percentage of positive cells, using a semi-quantitative immunoreactivity score system. Samples with strong membrane or nuclear staining in over 50% of tumor cells were classified as high expression, while those with weak or focal staining were considered low expression.

### Statistical analysis

Bioinformatics analysis was conducted using R software (version 3.6.0). Experimental data were analyzed using SPSS 25.0 software, and graphs were generated with GraphPad Prism 8. Data were presented as mean ± standard deviation or median as appropriate. Survival analyses were conducted using the Kaplan-Meier method and the log-rank test. The inter-group differences were evaluated via unpaired Student’s t-test (for two groups) or one-way ANOVA with Tukey’s post hoc test (for multiple groups). A *P*-value < 0.05 was considered statistically significant.

## Supplementary information

Table S1

Table S2

Fig. S1

Supplementary Methods and Fig. Legends

Uncropped western blots

## Data Availability

Data is provided within the manuscript or supplementary information files.
