# Supplementary material for: PIN1 enhances SUMOylation of YAP1 and inhibits ferroptosis via autophagy-dependent degradation of ACSL4 in cervical cancer
Source: Cell Death Discov. 2026 Jul 22;12:418. doi: 10.1038/s41420-026-03257-x (PMC13672308; doi:10.1038/s41420-026-03257-x)
Supplement: Supplementary file 1 [file 41420_2026_3257_MOESM5_ESM.pdf]

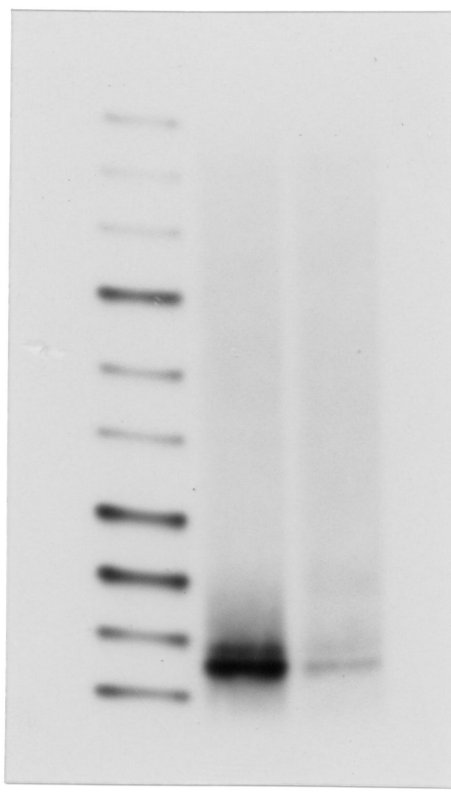

Figure legend. The western blot results of PIN1 protein in HeLa cells among the sh-NC, sh-PIN1 groups.

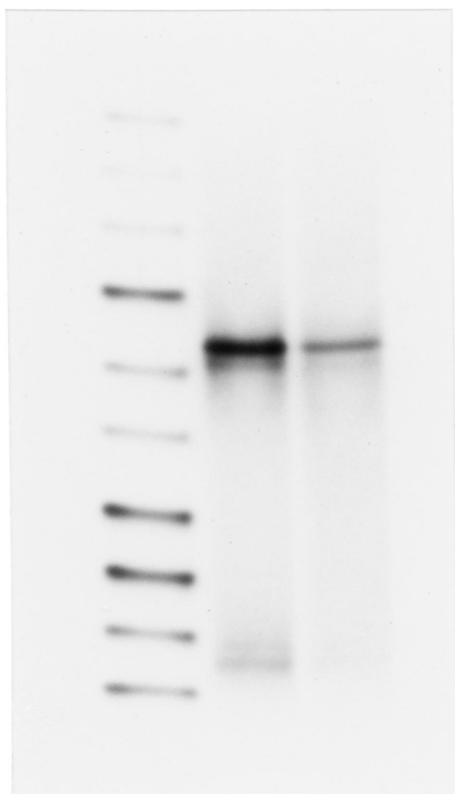

Figure legend. The western blot results of YAP1 protein in HeLa cells among the sh-NC, sh-PIN1 groups.

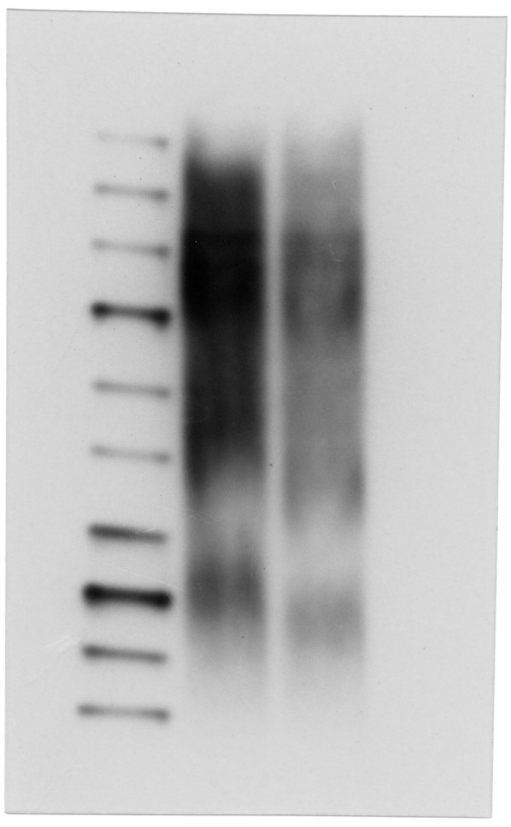

Figure legend. The western blot results of SUMO1 protein in HeLa cells among the sh-NC, sh-PIN1 groups.

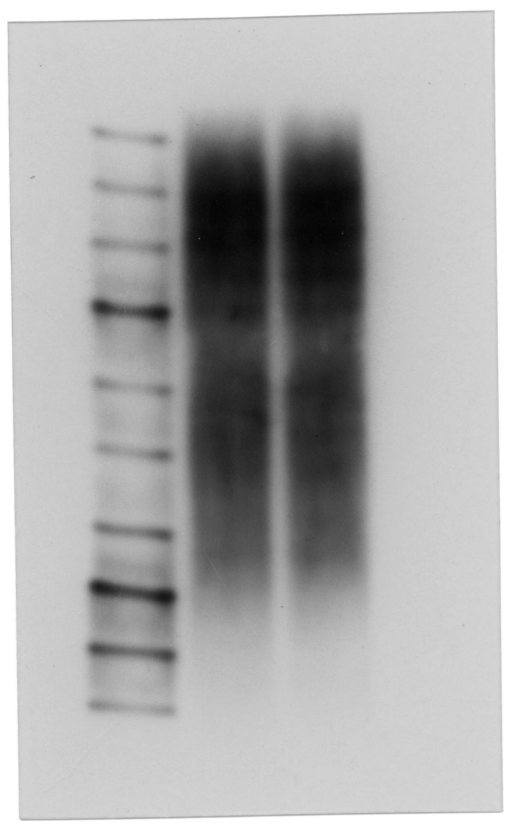

Figure legend. The western blot results of SUMO2/3 protein in HeLa cells among the sh-NC, sh-PIN1 groups.

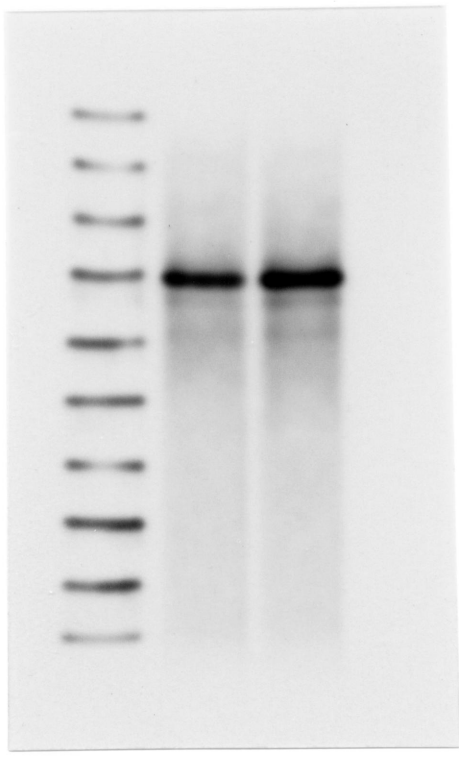

Figure legend. The western blot results of ACSL4 protein in HeLa cells among the sh-NC, sh-PIN1 groups.

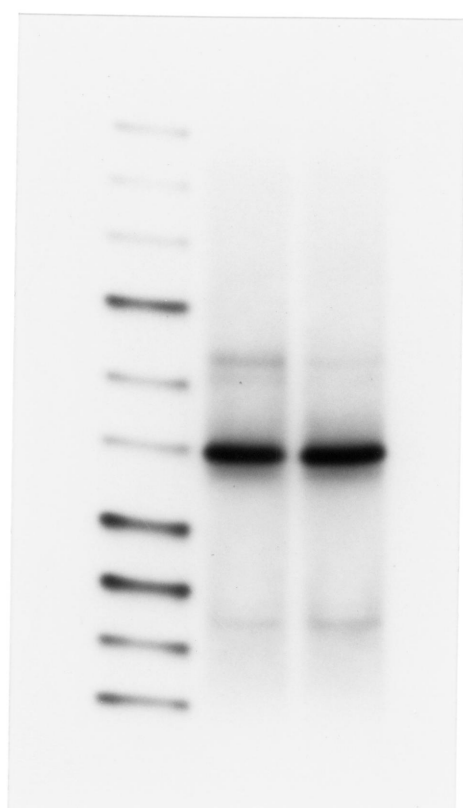

Figure legend. The western blot results of Tubulin protein in HeLa cells among the sh-NC, sh-PIN1 groups.

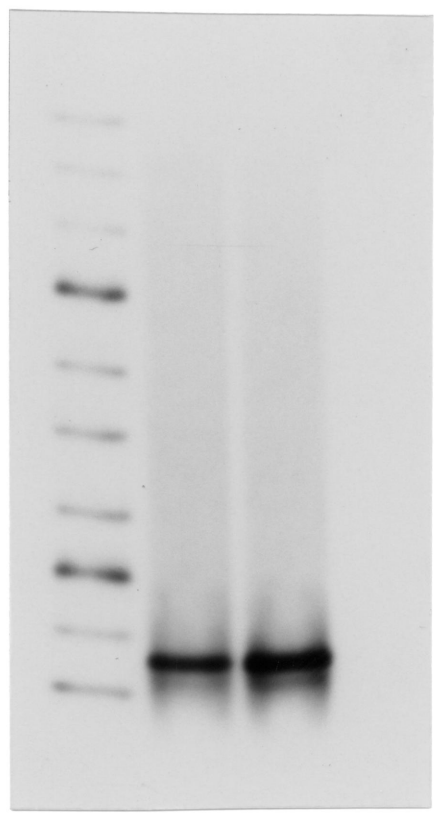

Figure legend. The western blot results of PIN1 protein in SiHa cells among the OE-NC, OE-PIN1 groups.

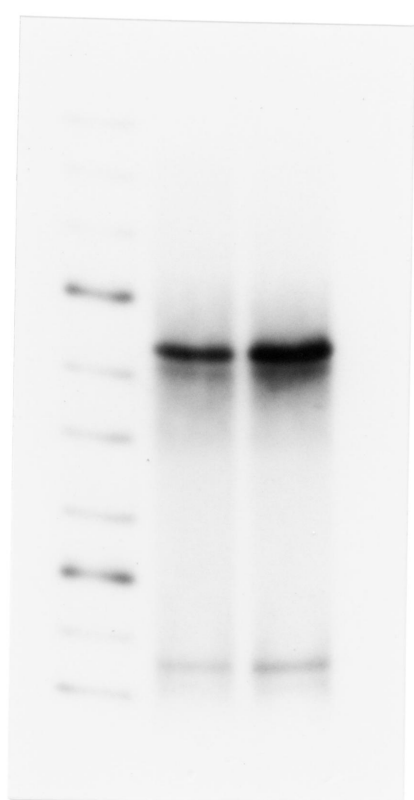

Figure legend. The western blot results of YAP1 protein in SiHa cells among the OE-NC, OE-PIN1 groups.

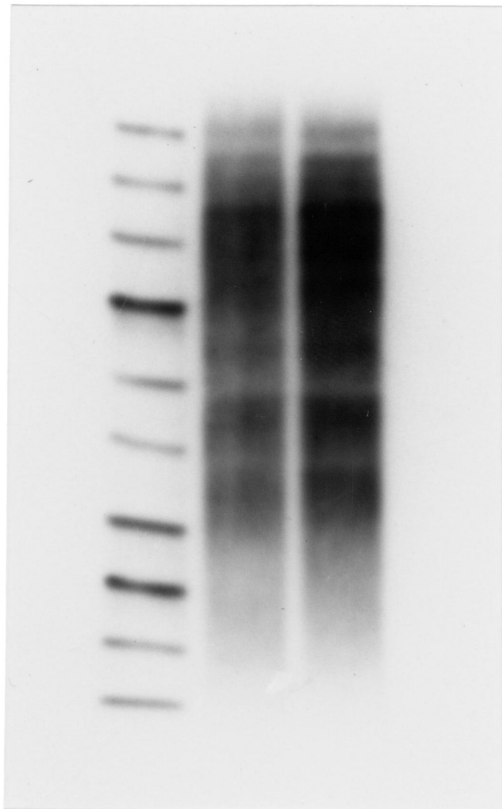

Figure legend. The western blot results of SUMO1 protein in SiHa cells among the OE-NC, OE-PIN1 groups.

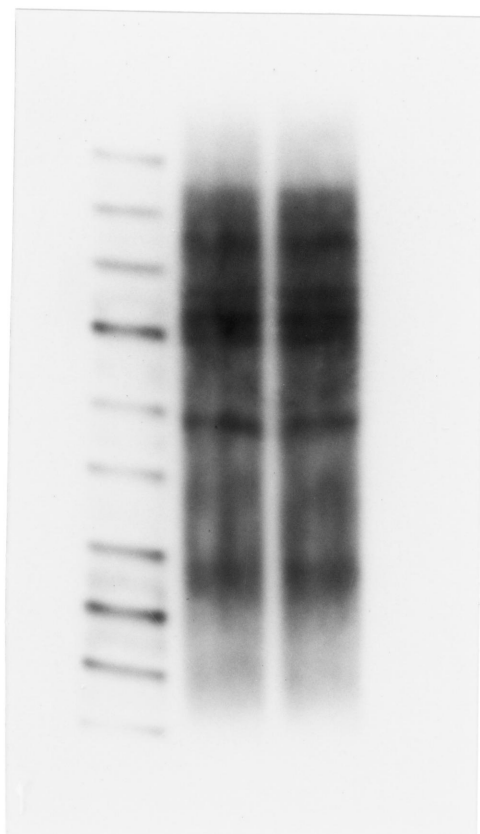

Figure legend. The western blot results of SUMO2/3 protein in SiHa cells among the OE-NC, OE-PIN1 groups.

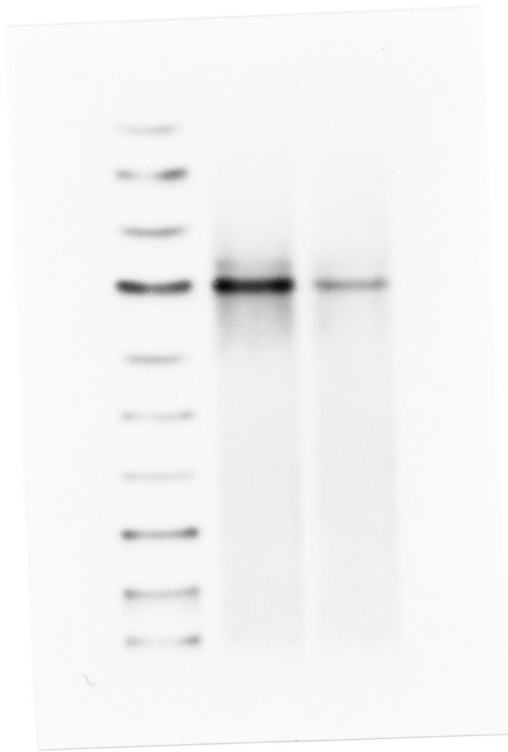

Figure legend. The western blot results of ACSL4 protein in SiHa cells among the OE-NC, OE-PIN1 groups.

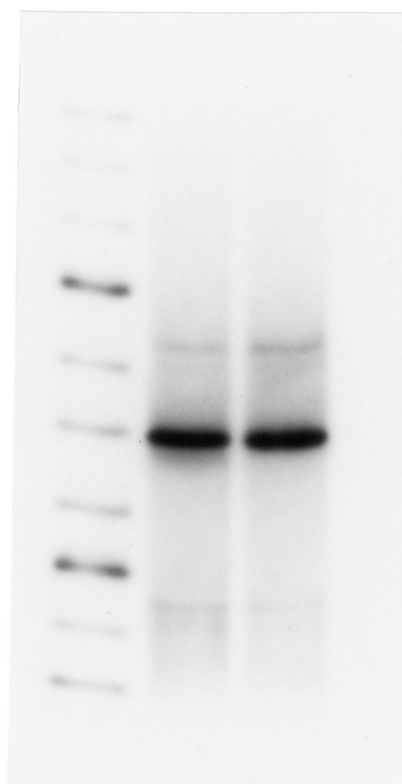

Figure legend. The western blot results of Tubulin protein in SiHa cells among the OE-NC, OE-PIN1 groups.

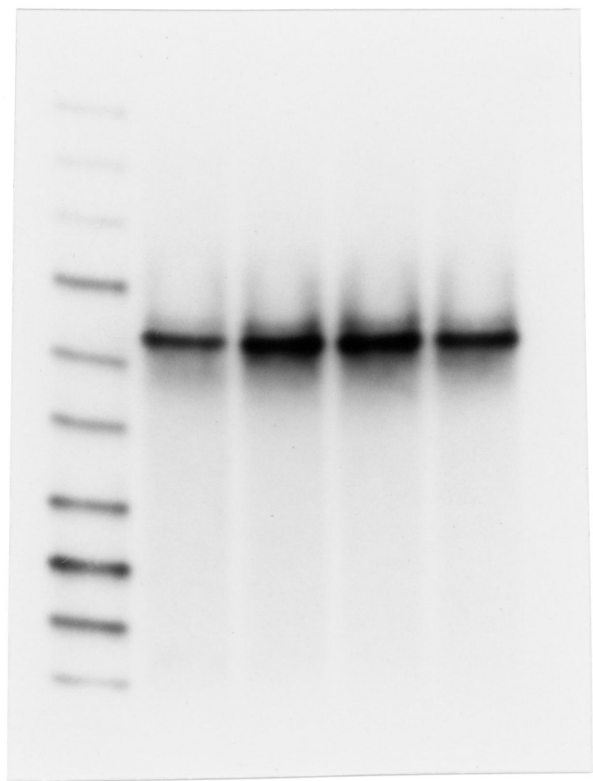

Figure legend. The western blot results of YAP1 protein in HeLa cells among the Control, N106, N106 + sh-NC, N106 + sh-PIN1 groups.

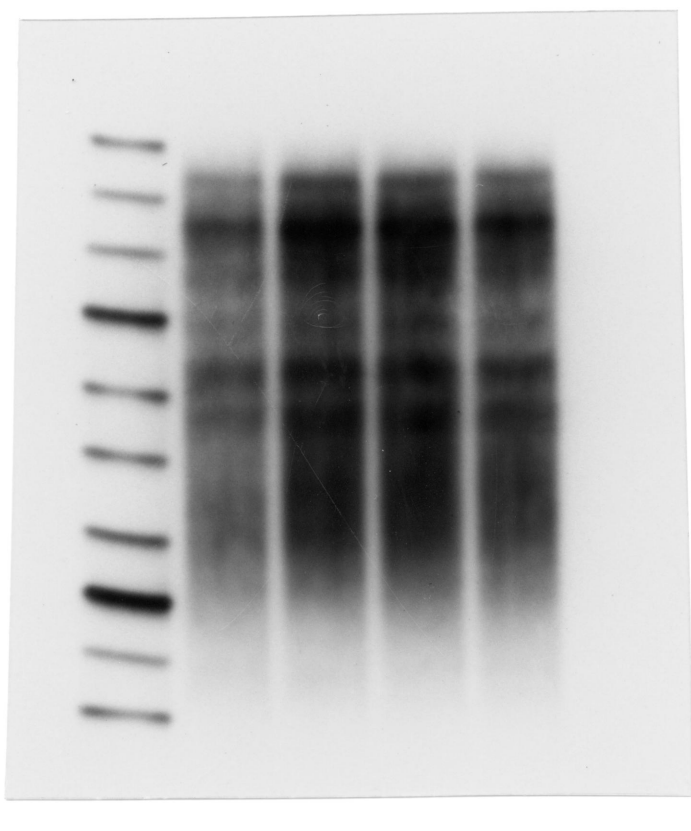

Figure legend. The western blot results of SUMO1 protein in HeLa cells among the Control, N106, N106 + sh-NC, N106 + sh-PIN1 groups.

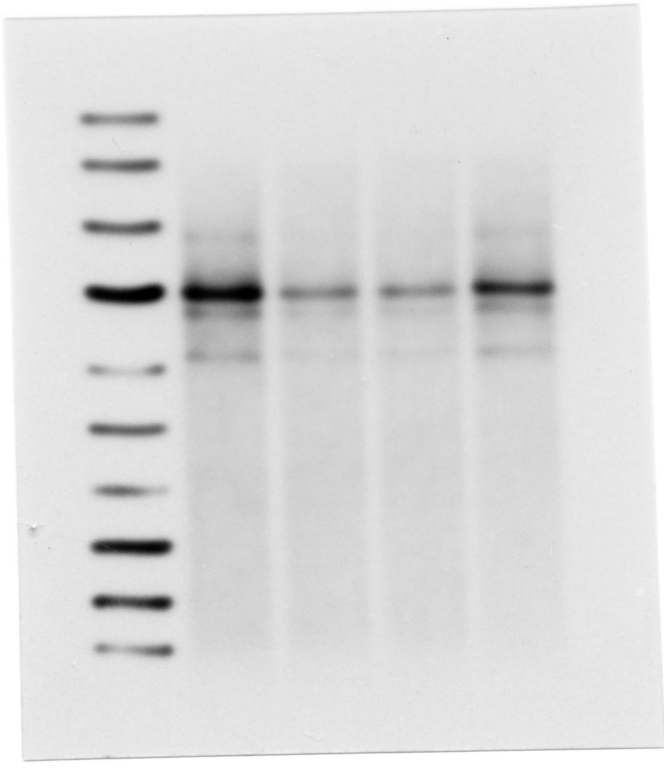

Figure legend. The western blot results of ACSL4 protein in HeLa cells among the Control, N106, N106 + sh-NC, N106 + sh-PIN1 groups.

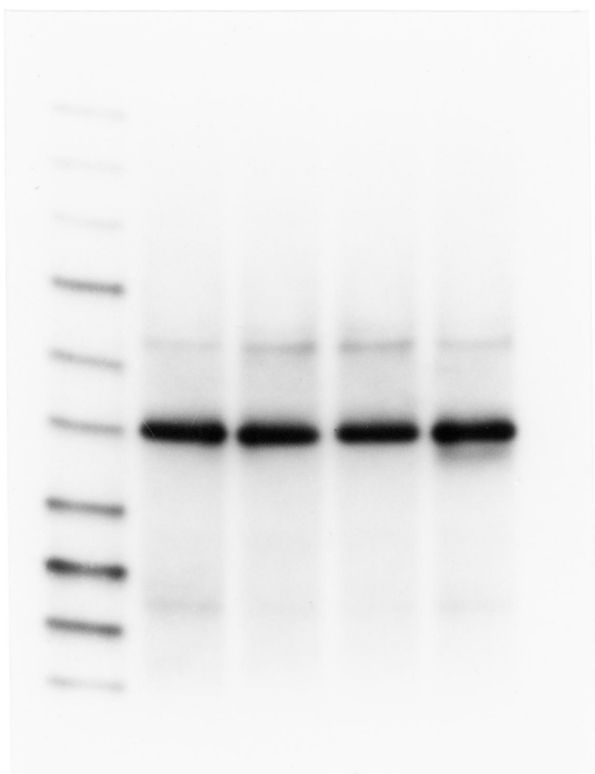

Figure legend. The western blot results of Tubulin protein in HeLa cells among the Control, N106, N106 + sh-NC, N106 + sh-PIN1 groups.

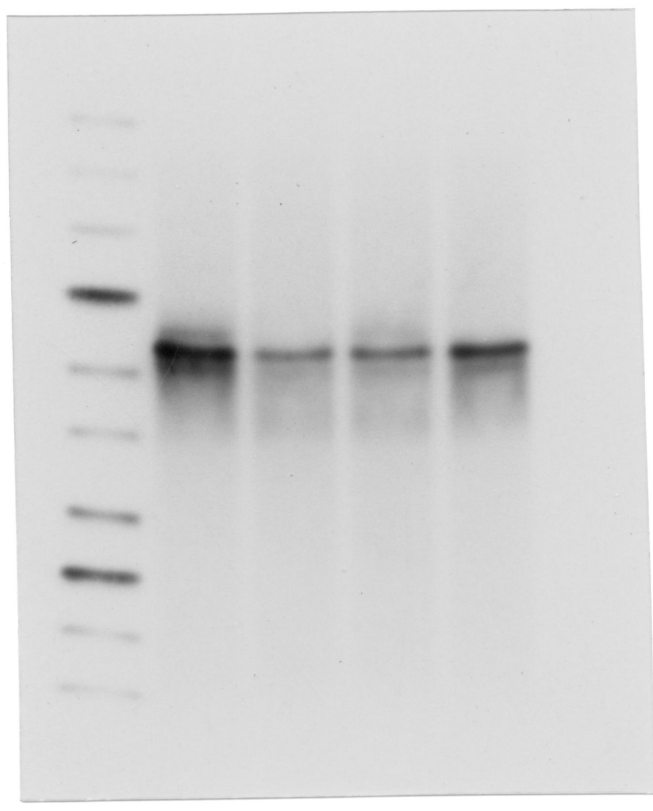

Figure legend. The western blot results of YAP1 protein in SiHa cells among the Control, TAK-981, TAK-981 + OE-NC, TAK-981 + OE-PIN1 groups.

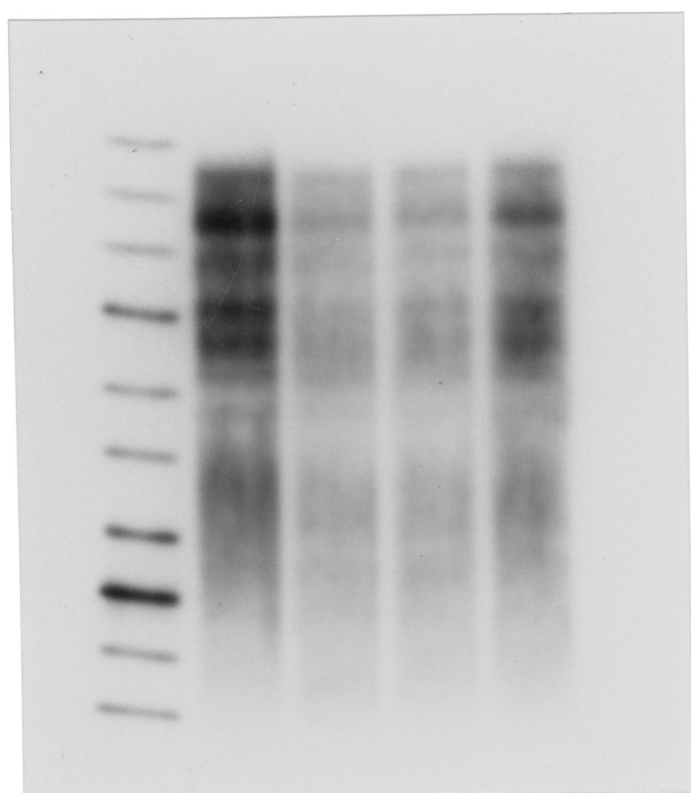

Figure legend. The western blot results of SUMO1 protein in SiHa cells among the Control, TAK-981, TAK-981 + OE-NC, TAK-981 + OE-PIN1 groups.

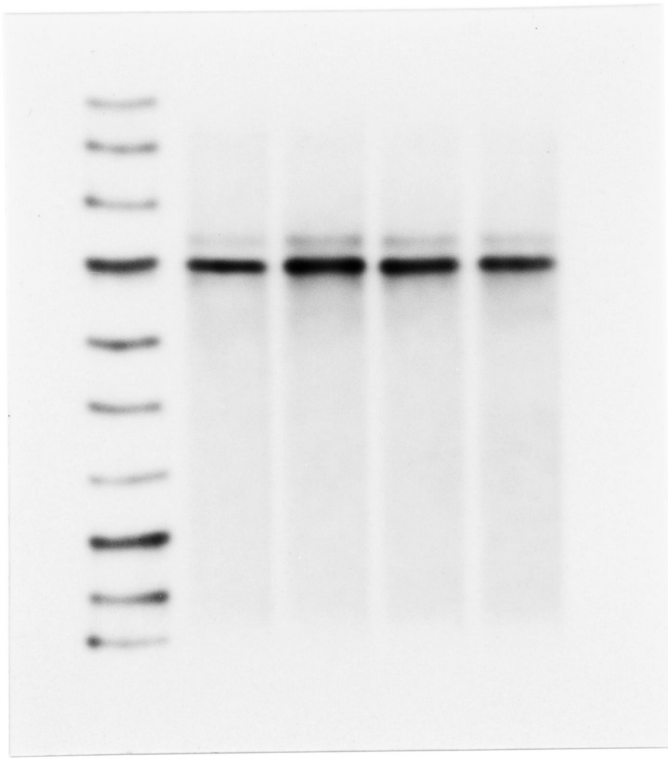

Figure legend. The western blot results of ACSL4 protein in SiHa cells among the Control, TAK-981, TAK-981 + OE-NC, TAK-981 + OE-PIN1 groups.

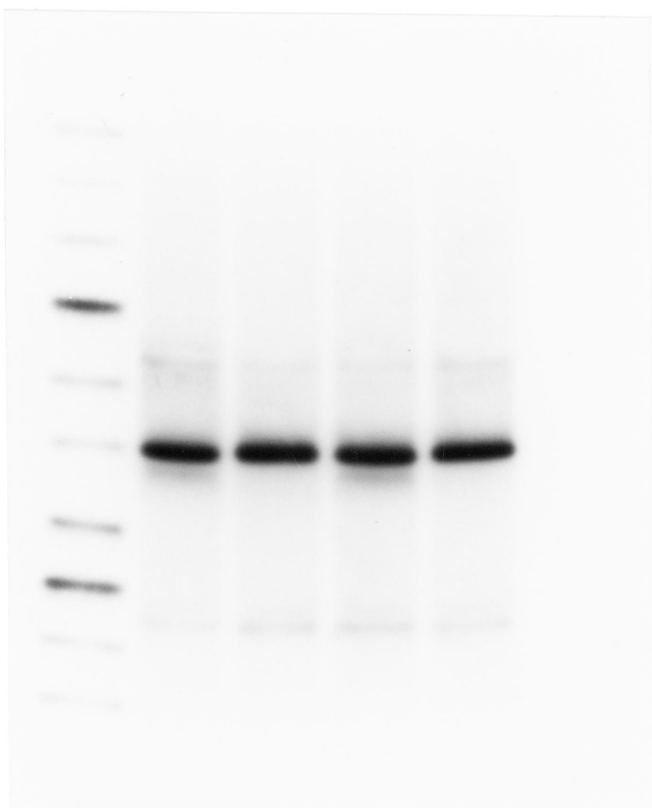

Figure legend. The western blot results of Tubulin protein in SiHa cells among the Control, TAK-981, TAK-981 + OE-NC, TAK-981 + OE-PIN1 groups.

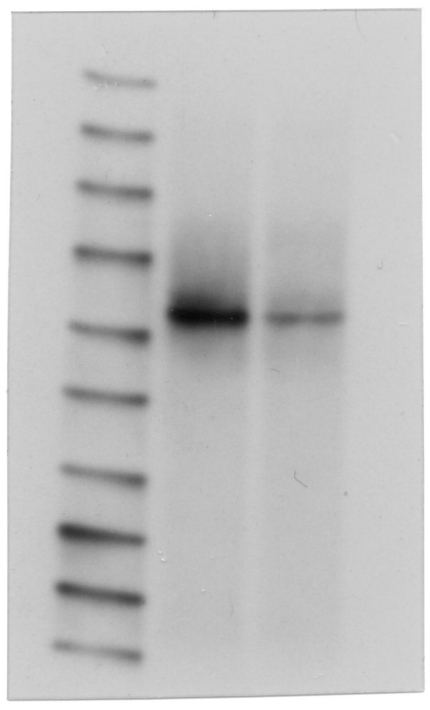

Figure legend. The western blot results of YAP1 protein in HeLa cells among the sh-NC, sh-YAP1 groups.

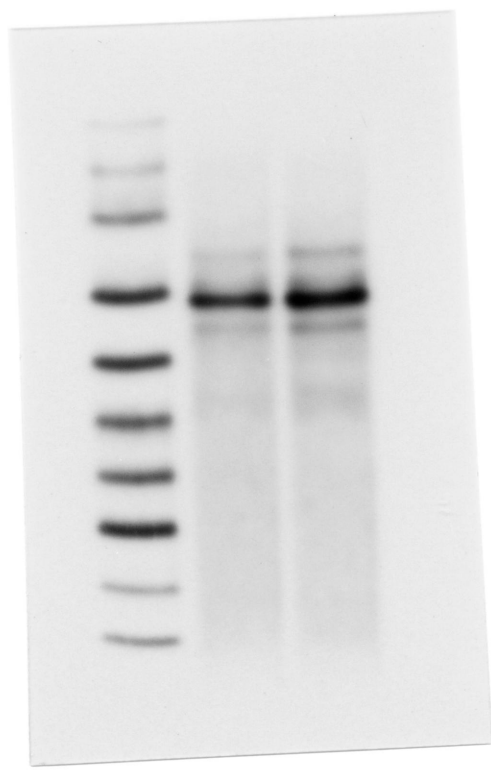

Figure legend. The western blot results of ACSL4 protein in HeLa cells among the sh-NC, sh-YAP1 groups.

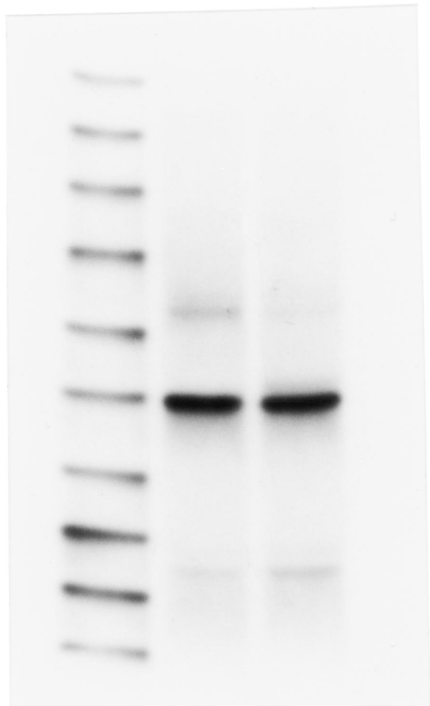

Figure legend. The western blot results of Tubulin protein in HeLa cells among the sh-NC, sh-YAP1 groups.

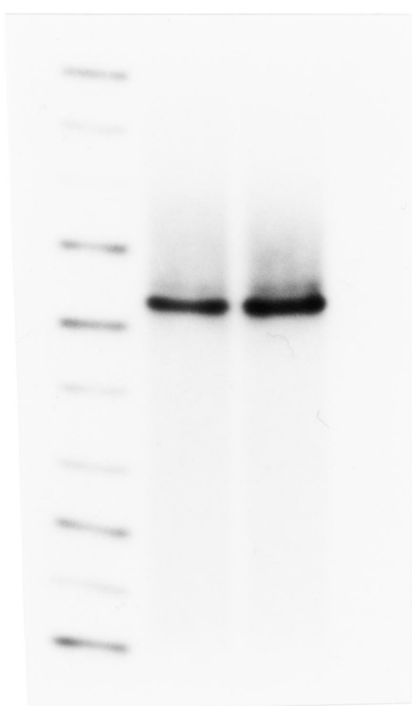

Figure legend. The western blot results of YAP1 protein in SiHa cells among the OE-NC, OE-YAP1 groups.

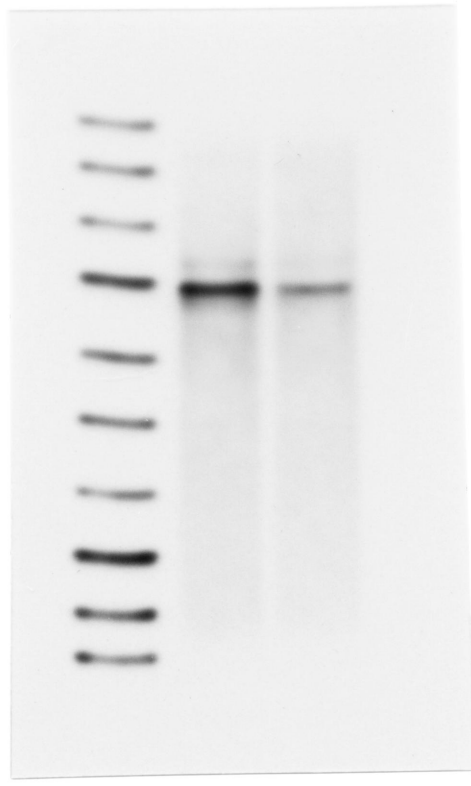

Figure legend. The western blot results of ACSL4 protein in SiHa cells among the OE-NC, OE-YAP1 groups.

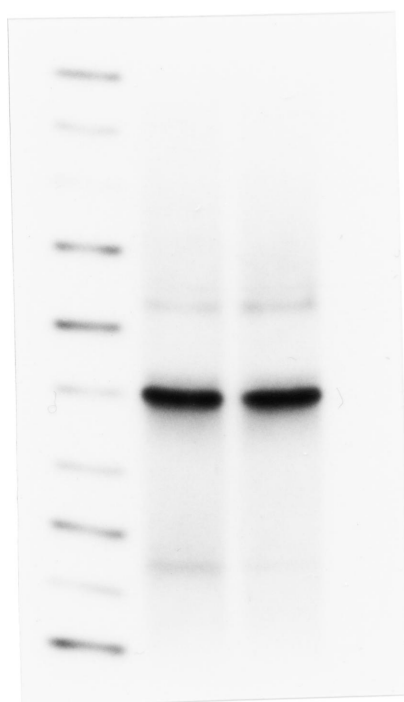

Figure legend. The western blot results of Tubulin protein in SiHa cells among the OE-NC, OE-YAP1 groups.

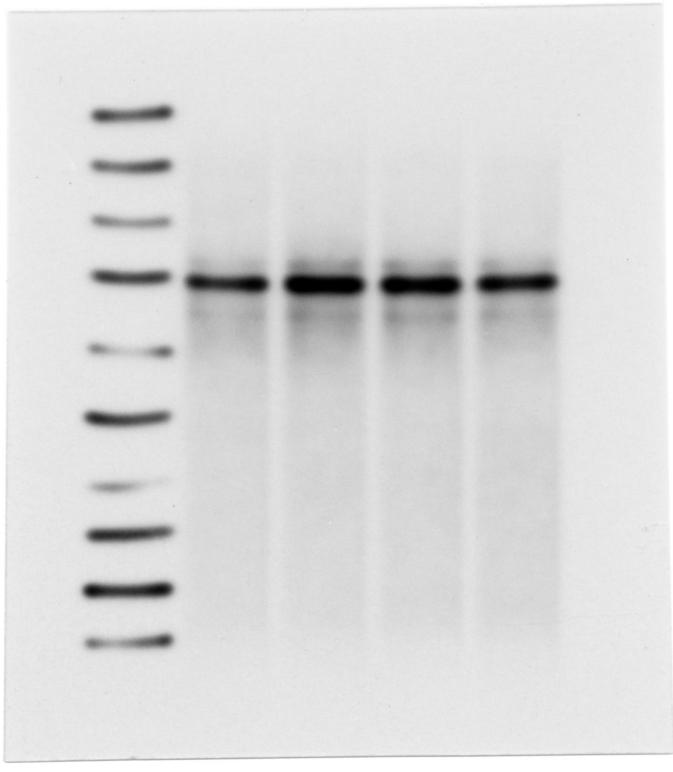

Figure legend. The western blot results of ACSL4 protein in SiHa cells among the Control, Bafilomycin A1, Bafilomycin A1 + OE-NC, Bafilomycin A1 + OE-YAP1 groups.

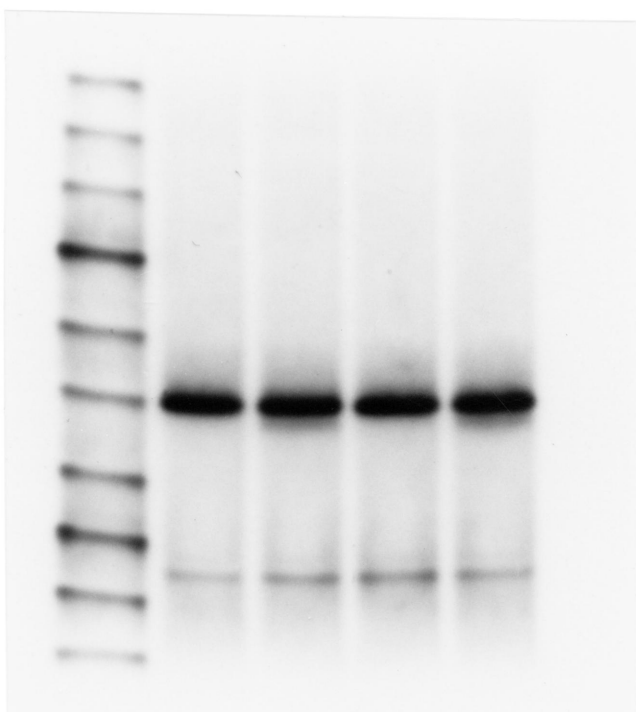

Figure legend. The western blot results of Tubulin protein in SiHa cells among the

Control, Bafilomycin A1, Bafilomycin A1 + OE-NC, Bafilomycin A1 + OE-YAP1 groups.

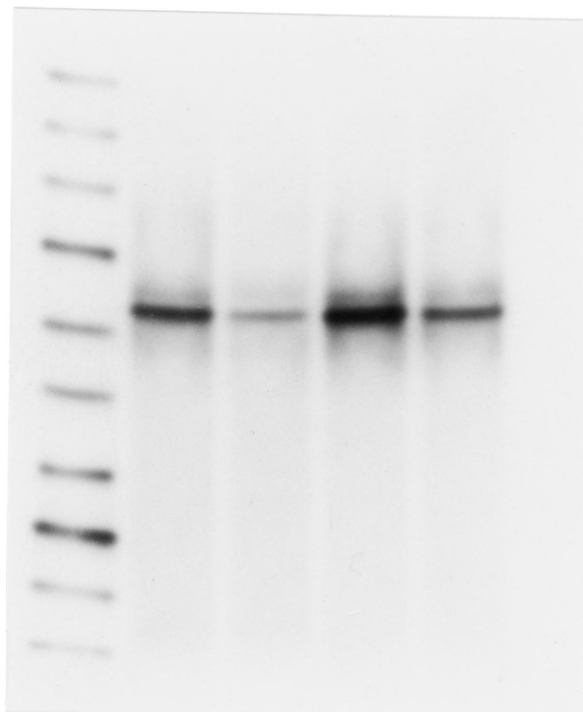

Figure legend. The western blot results of YAP1 protein in tumour tissues among the sh-NC, sh-PIN1, N106 + sh-NC, N106 + sh-PIN1 groups.

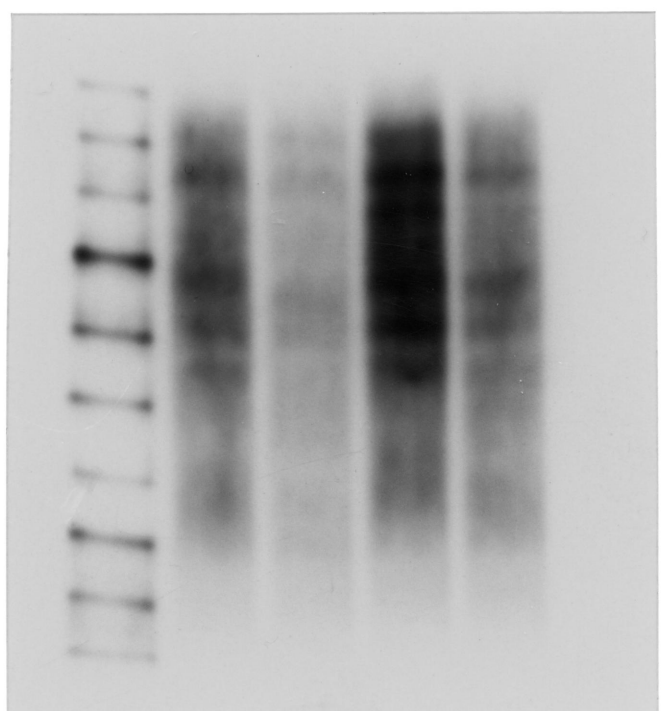

Figure legend. The western blot results of SUMO1 protein in tumour tissues among the sh-NC, sh-PIN1, N106 + sh-NC, N106 + sh-PIN1 groups.

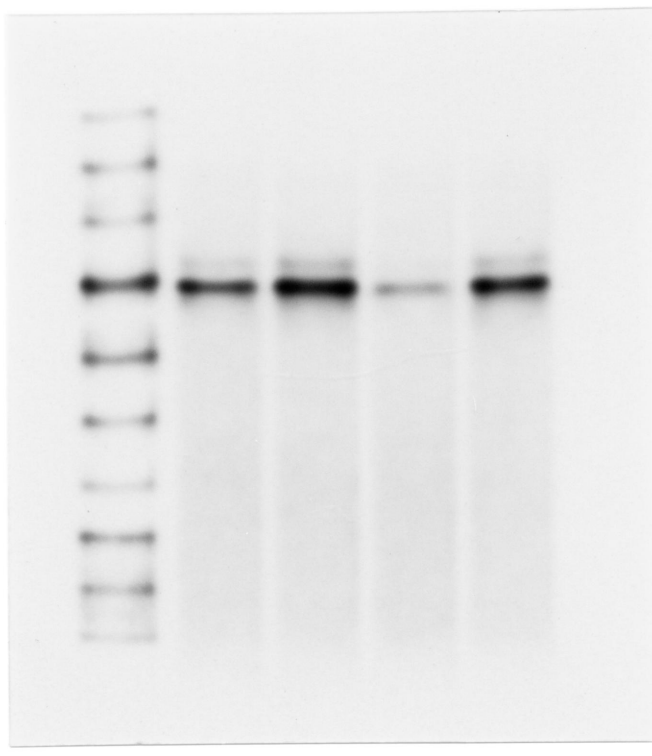

Figure legend. The western blot results of ACSL4 protein in tumour tissues among the sh-NC, sh-PIN1, N106 + sh-NC, N106 + sh-PIN1 groups.

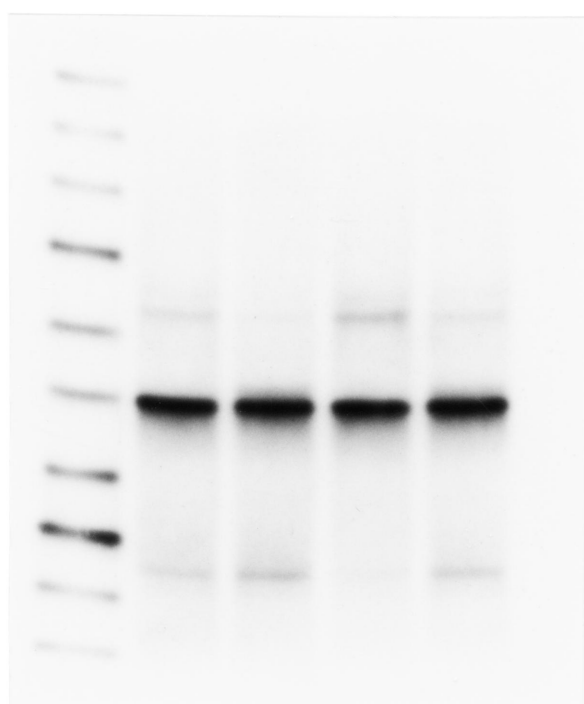

Figure legend. The western blot results of Tubulin protein in tumour tissues among the sh-NC, sh-PIN1, N106 + sh-NC, N106 + sh-PIN1 groups.

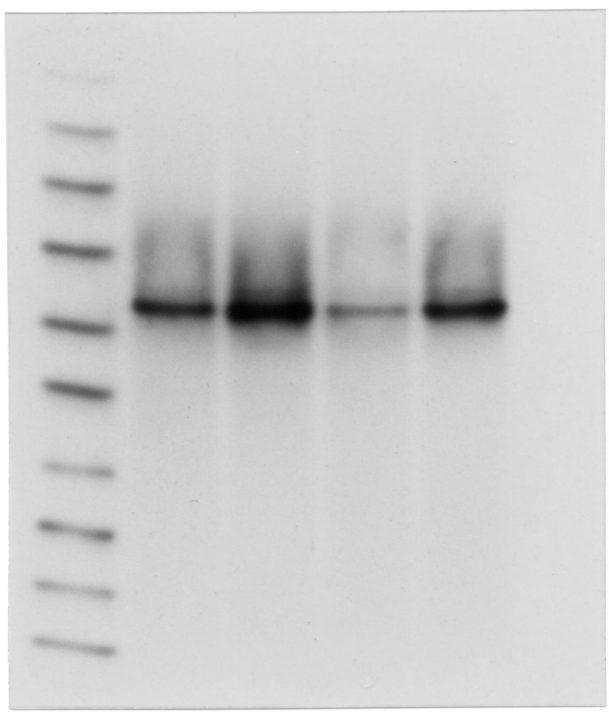

Figure legend. The western blot results of YAP1 protein in tumour tissues among the OE-NC, OE-PIN1, TAK-981 + OE-NC, TAK-981 + OE-PIN1 groups.

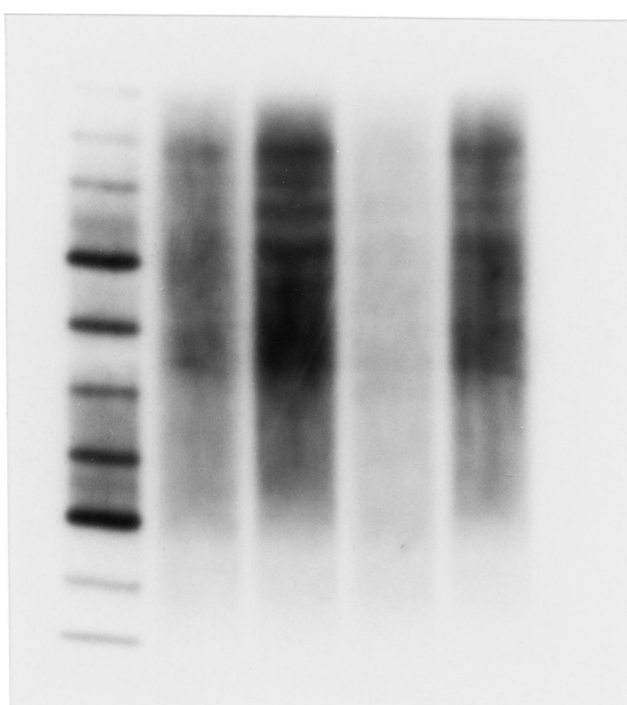

Figure legend. The western blot results of SUMO1 protein in tumour tissues among the OE-NC, OE-PIN1, TAK-981 + OE-NC, TAK-981 + OE-PIN1 groups.

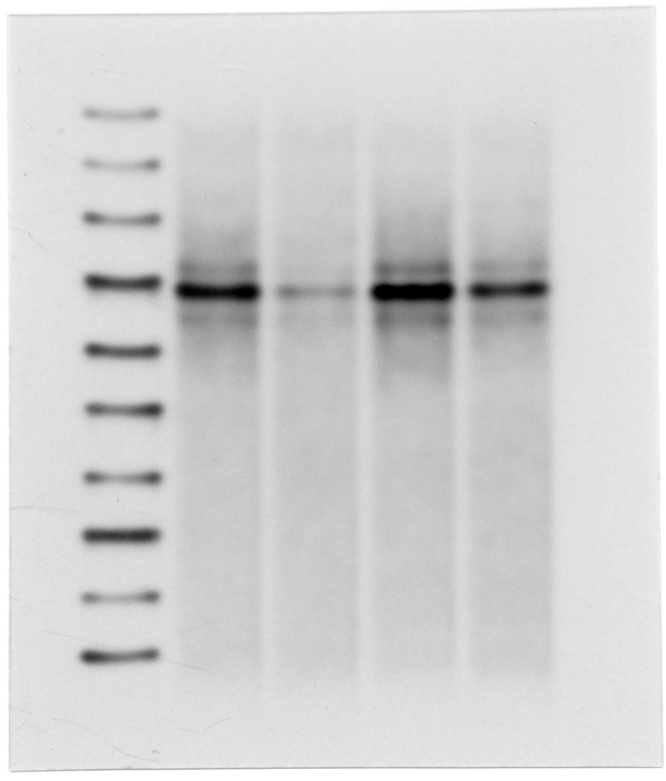

Figure legend. The western blot results of ACSL4 protein in tumour tissues among the OE-NC, OE-PIN1, TAK-981 + OE-NC, TAK-981 + OE-PIN1 groups.

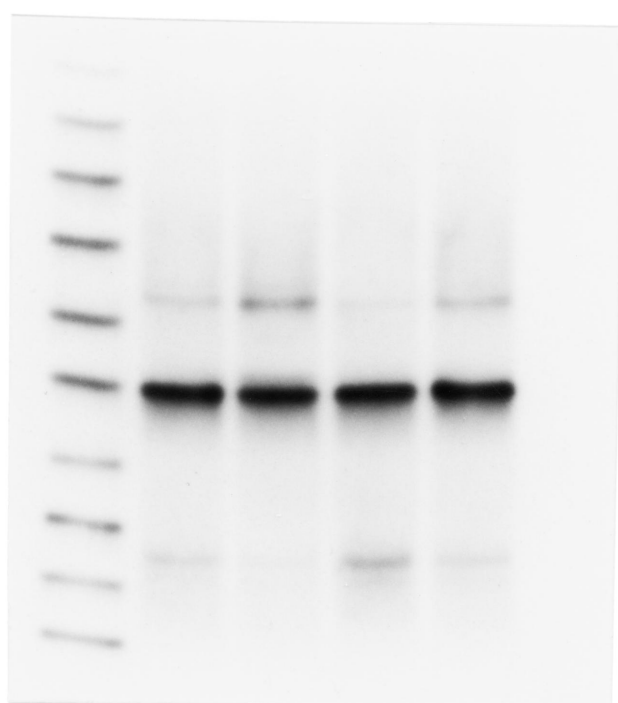

Figure legend. The western blot results of Tubulin protein in tumour tissues among the OE-NC, OE-PIN1, TAK-981 + OE-NC, TAK-981 + OE-PIN1 groups.

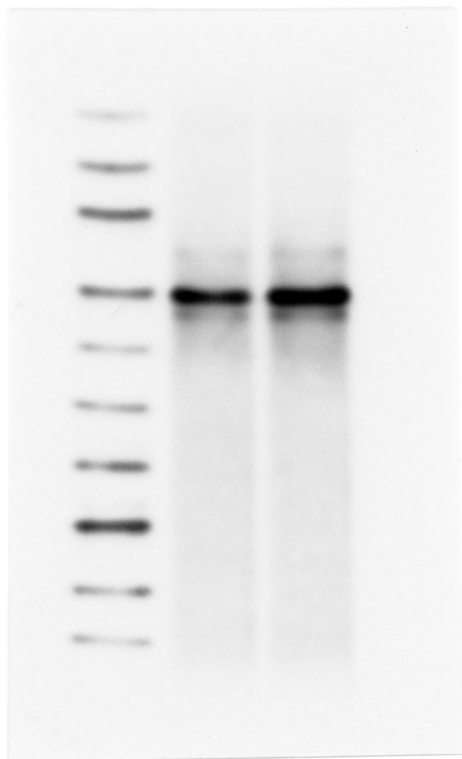

Figure legend. The western blot results of ACSL4 protein in tumour tissues among the sh-NC, sh-YAP1 groups.

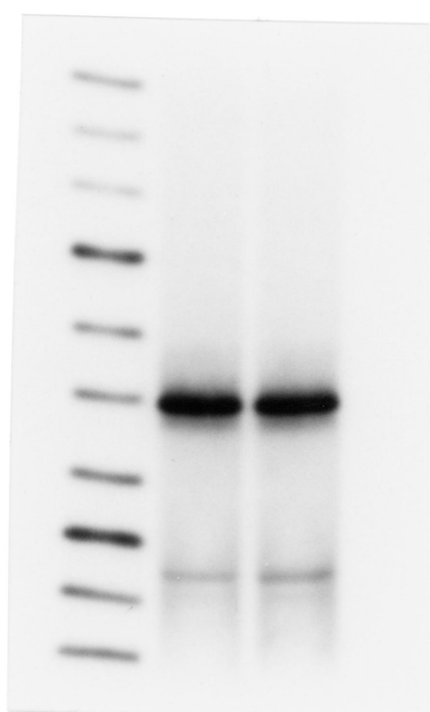

Figure legend. The western blot results of Tubulin protein in tumour tissues among the sh-NC, sh-YAP1 groups.

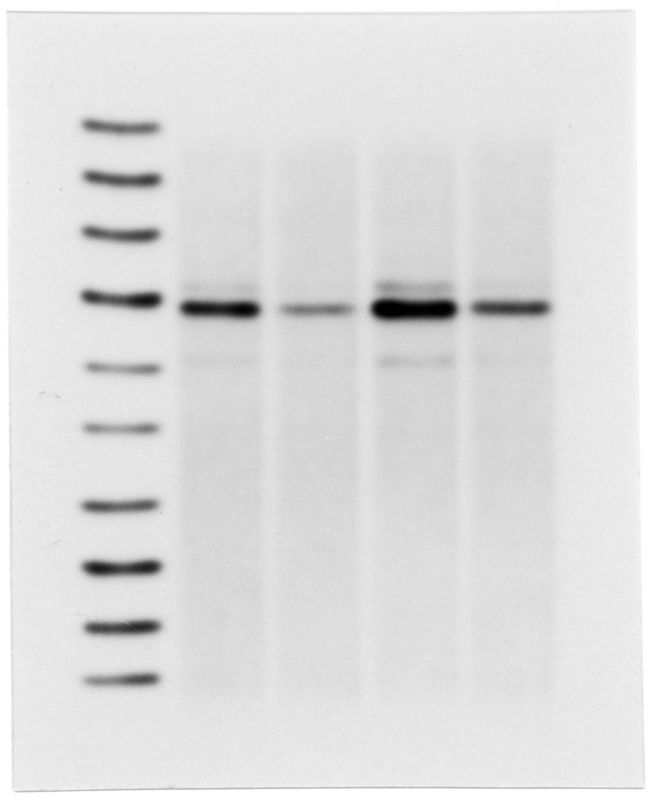

Figure legend. The western blot results of ACSL4 protein in tumour tissues among the OE-NC, OE-YAP1, Bafilomycin A1 + OE-NC, Bafilomycin A1 + OE-YAP1 groups.

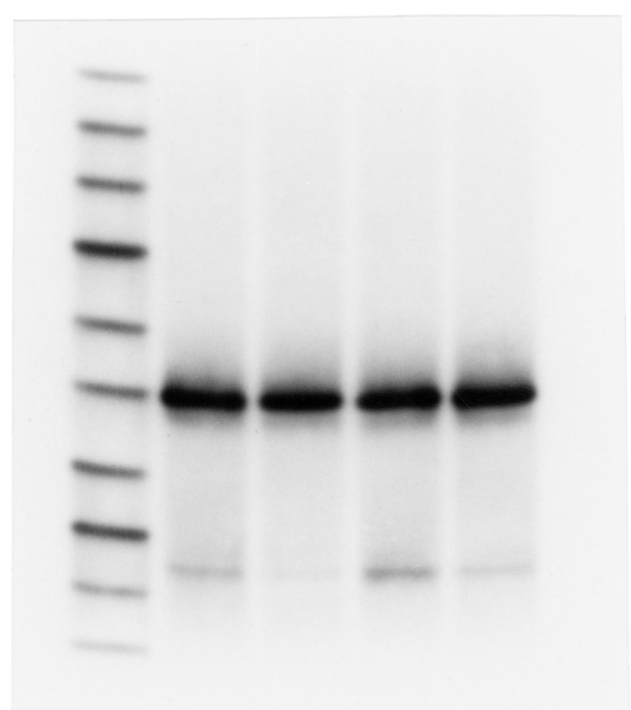

Figure legend. The western blot results of Tubulin protein in tumour tissues among the OE-NC, OE-YAP1, Bafilomycin A1 + OE-NC, Bafilomycin A1 + OE-YAP1 groups.
