# Supplementary material for: PIN1 enhances SUMOylation of YAP1 and inhibits ferroptosis via autophagy-dependent degradation of ACSL4 in cervical cancer
Source: Cell Death Discov. 2026 Jul 22;12:418. doi: 10.1038/s41420-026-03257-x (PMC13672308; doi:10.1038/s41420-026-03257-x)
Supplement: Supplementary file 2 [file 41420_2026_3257_MOESM1_ESM.docx]

**Table S1.** The sequences for PIN1 and YAP1 knockdown and overexpression.

| Name | sense 5’-3’ | antisense 5’-3’ |
| --- | --- | --- |
| sh-PIN1-1 | CCGGCCAGAAGATCAAGTCGGGAGACTCGAGTCTCCCGACTTGATCTTCTGGTTTTT | AATTAAAAACCAGAAGATCAAGTCGGGAGACTCGAGTCTCCCGACTTGATCTTCTGG |
| sh-PIN1-2 | CCGGAGGAGAAGATCACCCGGACCACTCGAGTGGTCCGGGTGATCTTCTCCTTTTTT | AATTAAAAAAGGAGAAGATCACCCGGACCACTCGAGTGGTCCGGGTGATCTTCTCCT |
| sh-PIN1-3 | CCGGGCCATTTGAAGACGCCTCGTTCTCGAGAACGAGGCGTCTTCAAATGGCTTTTT | AATTAAAAAGCCATTTGAAGACGCCTCGTTCTCGAGAACGAGGCGTCTTCAAATGGC |
| OE-PIN1 | TACCGAGCTCGGATCCGCCACCATGGCGGACGAGGAGAAG | GATATCTGCAGAATTCTCACTCAGTGCGGAGGATG |
| sh-YAP1-1 | CCGGGGTCAGAGATACTTCTTAAATCTCGAGATTTAAGAAGTATCTCTGACCTTTTT | AATTAAAAAGGTCAGAGATACTTCTTAAATCTCGAGATTTAAGAAGTATCTCTGACC |
| sh-YAP1-2 | CCGGGGTGATACTATCAACCAAAGCCTCGAGGCTTTGGTTGATAGTATCACCTTTTT | AATTAAAAAGGTGATACTATCAACCAAAGCCTCGAGGCTTTGGTTGATAGTATCACC |
| sh-YAP1-3 | CCGGCGTCCAGCAAGATACTTTAATCTCGAGATTAAAGTATCTTGCTGGACGTTTTT | AATTAAAAACGTCCAGCAAGATACTTTAATCTCGAGATTAAAGTATCTTGCTGGACG |
| OE-YAP1 | AATGTACAAGGAATTCGCCACCATGGATCCCGGGCAGCAG | AATTATCTAGGGATCCCTATAACCATGTAAGAAAGCTTTC |
