# Supplementary material for: PIN1 enhances SUMOylation of YAP1 and inhibits ferroptosis via autophagy-dependent degradation of ACSL4 in cervical cancer
Source: Cell Death Discov. 2026 Jul 22;12:418. doi: 10.1038/s41420-026-03257-x (PMC13672308; doi:10.1038/s41420-026-03257-x)
Supplement: Supplementary file 3 [file 41420_2026_3257_MOESM2_ESM.docx]

**Table S2.** Primers for qRT-PCR.

| Gene | Forward | Reverse |
| --- | --- | --- |
| PIN1 | TCAGGCCGAGTGTACTACTTC | TCTTCTGGATGTAGCCGTTGA |
| YAP1 | TAGCCCTGCGTAGCCAGTTA | TCATGCTTAGTCCACTGTCTGT |
| GAPDH | GGAGCGAGATCCCTCCAAAAT | GGCTGTTGTCATACTTCTCATGG |
