# Supplementary material for: PIN1 enhances SUMOylation of YAP1 and inhibits ferroptosis via autophagy-dependent degradation of ACSL4 in cervical cancer
Source: Cell Death Discov. 2026 Jul 22;12:418. doi: 10.1038/s41420-026-03257-x (PMC13672308; doi:10.1038/s41420-026-03257-x)
Supplement: Supplementary file 4 [file 41420_2026_3257_MOESM4_ESM.docx]

# **Supplementary Methods:**

**Differential Expression Analysis:** We conducted differential expression analysis of relevant genes using the TCGA-CESC database, which included 309 tumor samples and 3 adjacent non-tumor samples.

**KM Curve Analysis:** We screened relevant samples from the TCGA-CESC database, excluded adjacent non-tumor samples and those without clinical information, and obtained a total of 307 valid samples. The samples were grouped based on the median expression levels of the genes and analyzed using the R package “survival”.

**ROC Curve Analysis:** We evaluated the overall survival (OS) probability and prognostic differences using 307 valid samples with the timeROCR package

**Correlation Analysis:** Using the TCGA-CESC database, the Pearson method was employed to assess the correlation between two variables

**Molecular Docking:** Download experimentally determined protein structure files from the PDB database (https://www.rcsb.org/). Process the protein structures by removing water molecules and heteroatoms (e.g., ions). Add hydrogen atoms and Gasteiger charges. Finally, execute the Autodock docking program to generate binding conformations and binding energy results.

# **Supplementary Figure Legends：**

**Figure S1. PIN1 and YAP1 served as potential biomarkers for CC, and the PIN1-YAP1 axis may regulate the expression of ACSL4, which served as a biomarker for ferroptosis.**

(A) Differential expression analysis of PIN1 in cervical cancer tissue and normal tissue. (B) KM analysis results of PIN1 in cervical cancer. (C) ROC results of PIN1 in cervical cancer. (D) Correlation analysis between PIN1 and YAP1. (E) Molecular docking of PIN1 and YAP1. (F) Differential expression analysis of YAP1 in cervical cancer tissue and normal tissue. (G) KM analysis results of YAP1 in cervical cancer. (H) ROC results of YAP1 in cervical cancer. (I) Correlation analysis between YAP1 and ACSL4. (J) Correlation analysis between YAP1 and GPX4. (K) Correlation analysis between YAP1 and SLC7A11. ***P < 0.001.
